# The Interaction of BMP2‐Induced Defect Healing in Rat and Fixator Stiffness Modulates Matrix Alignment and Contraction

**DOI:** 10.1002/jbm4.10031

**Published:** 2018-04-17

**Authors:** Carolin Schwarz, Claus‐Eric Ott, Dag Wulsten, Erik Brauer, Sophie Schreivogel, Ansgar Petersen, Kerstin Hassanein, Linda Roewer, Tanja Schmidt, Bettina M Willie, Georg N Duda

**Affiliations:** ^1^ Julius Wolff Institute and Center for Musculoskeletal Surgery Charité − Universitätsmedizin Berlin corporate member of Freie Universität Berlin Humboldt‐Universität zu Berlin and Berlin Institute of Health Berlin Germany; ^2^ Berlin‐Brandenburg Center for Regenerative Therapies (BCRT) Charité − Universitätsmedizin Berlin corporate member of Freie Universität Berlin Humboldt‐Universität zu Berlin and Berlin Institute of Health Berlin Germany; ^3^ Institute for Medical Genetics and Human Genetics Charité − Universitätsmedizin Berlin corporate member of Freie Universität Berlin Humboldt‐Universität zu Berlin and Berlin Institute of Health Berlin Germany; ^4^ Research Group Development and Disease Max Planck Institute for Molecular Genetics Berlin Germany; ^5^ Research Center Shriners Hospitals for Children‐Canada Department of Pediatric Surgery McGill University Montreal Canada

**Keywords:** LARGE SEGMENTAL DEFECT, BMP2, FIXATOR STIFFNESS, MATRIX ALIGNMENT, CONTRACTION

## Abstract

Successful fracture healing requires a tight interplay between mechanical and biological cues. In vitro studies illustrated that mechanical loading modulates bone morphogenetic protein (BMP) signaling. However, in the early phases of large bone defect regeneration in vivo, the underlying mechanisms leading to this mechanosensation remained unknown. We investigated the interaction of BMP2 stimulation and mechanical boundary conditions in a rat critical‐sized femoral defect model (5 mm) stabilized with three distinctly different external fixator stiffness. Defects were treated with 5 μg rhBMP2 loaded on an absorbable collagen sponge. Early matrix alignment was monitored by second‐harmonic generation imaging. Bony bridging of defects and successive healing was monitored by histology at day 7 and day 14 as well as in vivo microCT at days 10, 21, and 42 post‐operation. Femora harvested at day 42 were characterized mechanically assessing torsional load to failure ex vivo. At tissue level, differences between groups were visible at day 14 with manifest bone formation in the microCT. Histologically, we observed prolonged chondrogenesis upon flexible fixation, whereas osteogenesis started earlier after rigid and semirigid fixation. At later time points, there was a boost of bone tissue formation upon flexible fixation, whereas other groups already displayed signs of tissue maturation. Based on gene expression profiling, we analyzed the mechanobiological interplay. Already at day 3, these analyses revealed differences in expression pattern, specifically of genes involved in extracellular matrix formation. Gene regulation correlating with fixator stiffness was pronounced at day 7 comprising genes related to immunological processes and cellular contraction. The influence of loading on matrix contraction was further investigated and confirmed in a 3D bioreactor. Taken together, we demonstrate an early onset of mechanical conditions influencing BMP2‐induced defect healing and shed light on gene regulatory networks associated with extracellular matrix organization and contraction that seemed to directly impact healing outcomes. © 2018 The Authors. *JBMR Plus* is published by Wiley Periodicals, Inc. on behalf of the American Society for Bone and Mineral Research.

## Introduction

Fracture healing is characterized by initial inflammation^1^ followed by a cascade of multiple processes guiding endochondral ossification and finally remodeling. Throughout this so‐called secondary bone healing, tissue is assembled in a way that allows it to carry increasing load. The self‐assembly process of callus tissue leads to incremental tissue stiffness and finally scar‐free intact bone.^2^, ^3^ The cascade of healing is seriously challenged in large defects where defect size extends bone width.

In these critical‐sized defects, bone morphogenetic proteins (BMPs) are frequently the only way to allow a reconstitution of bone integrity and salvage the limb. Current treatment relies on bone morphogenetic protein BMP2,^4^ eventually in combination with bone grafts or bone marrow aspirates.^5^ BMP2 is delivered with an absorbable collagen sponge in dosages ranging from 10 to 12 mg dependent on the patient's anamnesis and treatment site.^6^, ^7^, ^8^ Although BMP2 treatment in these dosages typically leads to successful healing, a series of preclinical and clinical studies have employed lower dosages to induce the bone self‐healing cascade.^9^, ^10^ Similarly, a reduced BMP2 dosage is quite efficient to induce successful healing of rat critical‐sized bone defects.^4^, ^11^

Adaption of cells to their biomechanical surrounding plays a critical role in wound healing and tissue regeneration.^12^, ^13^, ^14^, ^15^, ^16^ The initial healing phase is sensitive to mechanical conditions represented by the movement of the bone fragments. Type and magnitude of this so‐called interfragmentary movement determine vessel density in fibrous tissues and cascades of tissue differentiation.^17^, ^18^, ^19^ Recruitment of mesenchymal stromal cells and their differentiation are linked to mechanical stimuli.^2^, ^20^, ^21^, ^22^ Cells are directly interacting with the extracellular matrix (ECM) to sense mechanical forces.^12^, ^14^, ^23^ Cellular surface proteins transmit extracellular mechanical signals and lead to changes in cytoskeleton, intracellular signaling, and concomitant gene expression.^24^ Thus, extracellular mechanical conditions at a fracture site impact cellular function, shape, and ECM organization.

A direct interaction of BMP signaling and mechanically induced integrin signaling has been postulated in in vitro studies.^25^, ^26^ The interplay between mechanical and biological stimuli in vivo to gate either woven or lamellar bone formation has been demonstrated in mechanical loaded intact bone^27^, ^28^, ^29^ and distraction osteogenesis.^30^ Our group investigated this interaction in osteotomized bone.^31^ Additionally, we treated a rat critical‐sized defect with a high dose of BMP2 only and in combination with weekly controlled axial compressive mechanical loading. Combined treatment led to increased mineralized tissue formation and remodeling, but also significantly larger callus volume.^32^ Further studies showed that the mechanobiological interplay is already affected by physiological loading via weight bearing.^33^, ^34^, ^35^, ^36^ However, how mechanical conditions impact the BMP2‐induced bone formation in critical‐sized defect situations, specifically with respect to underlying molecular mechanisms, remained unknown so far.

**Figure 1:**
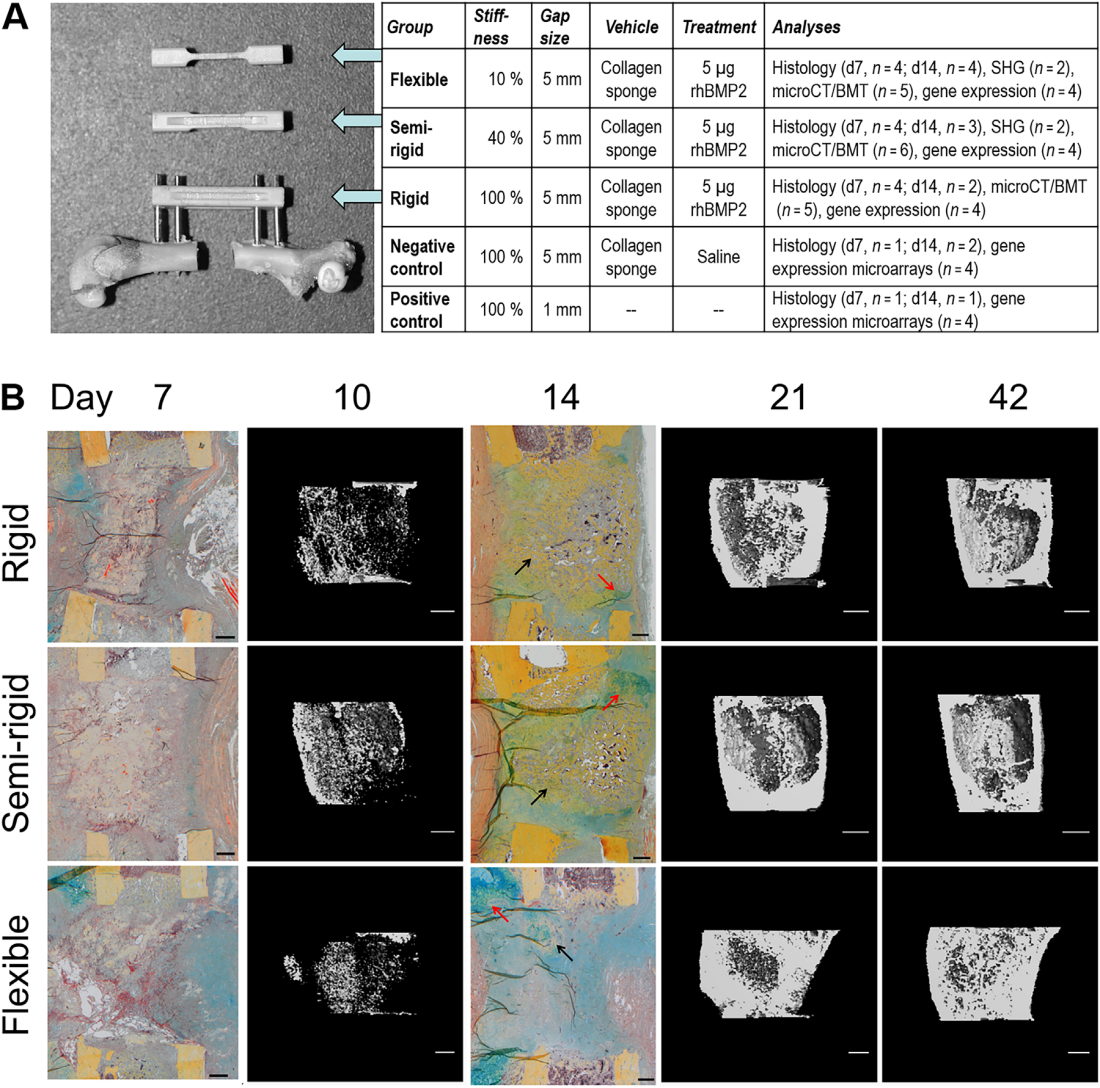
(*A*) Animal model. Defect model and sample overview. Rat femur with a 5‐mm defect gap and attached external fixator (RatExFix, RISystem) of rigid stability, accompanied by fixator bars of the semi‐rigid and flexible stabilities. Numbers of animals used for each analysis are given. SHG = second‐harmonic generation imaging; BMT = biomechanical testing. (*B*) Course of defect healing. Healing was similar in all groups at days 7 and 10. By day 14, the rigid and semi‐rigid group showed more woven bone (black arrows) at the defect site with islands of cartilage (red arrows). In contrast, at day 14, the flexible group showed less woven bone, with more proliferating cells across the gap and huge cartilage islands at periosteal site opposite to the fixator location. By day 21 and day 42, respectively, all groups revealed bony defect bridging (histology: Movat Pentachrome staining, scale bars = 500 μm; μCT: scale bars = 1 mm).

Here, we investigated the impact of 5 μg BMP2 in a critical‐sized 5‐mm defect in female rats accompanied with an external fixator system that allowed a subdivision into three different fixation stiffness groups (Fig. 1 *A*): rigid (100%), semi‐rigid (40% of rigid stability), and flexible (10% of rigid stability). According to an existing protocol,^37^ a nondestructive in vitro biomechanical torsional test was used to characterize the stability of the fixator constructs. The design of the rigid fixator resulted in a torsional stiffness of 6.12 ± 0.69 N/mm, the fixator design of the semi‐rigid fixator produced a torsional stiffness of 5.25 ± 1.55 N/mm, and the flexible variant achieved a torsional stiffness of 4.06 ± 0.49 N/mm (mean ± standard deviation). Control groups included an untreated 5‐mm defect loaded with a saline‐soaked absorbable collagen sponge as negative control, and a non‐critical‐sized 1‐mm osteotomy as positive control, both stabilized with the rigid fixator. We used gene expression analyses to investigate the regulatory networks involved in different healing outcomes. Most prominent biological processes included cellular contraction. Thus, we used a 3D bioreactor,^38^ which is well established in our lab, to confirm the impact of loading on volume contraction and scaffold stiffness.

## Materials and Methods

### Animal management

Female Sprague‐Dawley rats (12 weeks old, weight 240 to 320 g; Charles River, Sulzfeld, Germany) were ordered a minimum 1 week in advance and an acclimatization period was provided. Before surgery, animals were housed in groups of 5 in standard type 4 cages. Immediately after surgery, animals were single housed in type 3 cages until recovery, then pairwise until euthanization. Animal welfare, weight, gait pattern, and wound healing were controlled regularly. In all cages, gnawing and nesting material, tap water, and feed (standard rat diet, Sniff V1534‐000, ssniff Spezialdiäten GmbH, Soest, Germany) were provided *ad libitum*. Health monitoring followed the FELESA recommendations (SPF status).

### Animal surgery

Rats were anesthetized by intraperitoneal injection (Domitor, Pfizer, Karlsruhe, Germany; Ketamin, Actavis, München‐Riem, Germany). Before surgery, an antibiotic (Clindamycin, Ratiopharm, Ulm, Germany) and analgesic (Tramal Inject, Grünenthal, Aachen, Germany) were administered subcutaneously. Skin and fascia were incised in line with the left femur, which was further exposed by separating the *gluteus superficialis* and *biceps femoris* muscles. The first pin hole was made by hand close to the femoral distal epiphysis perpendicular to the femur axis. The connection element of the external fixator (RatExFix, RISystem AG, Davos, Switzerland)^39^ was attached to the femur with four locking screws to end up in an offset of 6 mm. To set a standardized double osteotomy in the femur diaphysis using an oscillating saw, a 5‐mm saw guide was fixed on the fixator bar. A clinically used absorbable collagen sponge (Lyostypt, B. Braun, Melsungen, Germany) was loaded either with saline or with 5 μg rhBMP2 (Walter Sebald, University of Würzburg, Würzburg, Germany) dissolved in low‐concentrated dilution buffer (4 mM hydrochloric acid) and placed into the defect. Wounds were closed (Vicryl 3‐0, Prolene 3‐0, Ethicon Inc., Somerville, NJ, USA) and postoperative analgesic supplied for 3 days with drinking water (Tramal drops, Grünenthal, Aachen, Germany). Animals were euthanized by intracardial injection of potassium chloride (B. Braun, Tuttlingen, Germany) under deep general anesthesia by intraperitoneal injection (Domitor, Pfizer, Karlsruhe, Germany; Ketamin, Actavis, München‐Riem, Germany). Animals were allocated randomly for treatment groups. The outcome of the study was unknown during allocation and animal handling, when assignment to experimental groups was obvious due to fixator design. All animal experiments were carried out according to the policies and procedures established by the Animal Welfare Act, the NIH Guide for Care and Use of Laboratory Animals, and the National Animal Welfare Guidelines. The study was approved by the local legal representative (Berlin LaGeSo G0248/11).

### Histology

At day 7 and day 14 post‐operation, animals were euthanized. Femora were fixed with 4% paraformaldehyde for 48 hours and decalcified in EDTA for 4 weeks. Samples were dehydrated in graded ethanol up to 100%, transferred to xylene, and embedded in paraffin. Four‐micron slides were cut in longitudinal direction and stained with Movat Pentachrome,^40^ indicating bone tissue in yellow, cartilage tissue in blue to green, and fibrous connective tissue in pink to purple.

### Micro‐computed tomography (μCT)

In vivo μCT was performed at days 10, 21, and 42 post‐operation (*n* = 5–6/group) (vivaCT 40, Scanco Medical, Brüttisellen, Switzerland; 55 kVp, 145 μA, and 150 ms integration time) according to published guidelines.^41^ The volume of interest included the 5‐mm osteotomy region. A global threshold of 50% of the mineral density of the intact limb, equivalent to 351 mg HA/ccm, was used to distinguish mineralized tissue (bone and calcified cartilage) from nonmineralized tissue. Outcome measures included mineralized callus volume (BV, mm^3^), total callus volume (TV, mm^3^), mineralized callus volume fraction (BV/TV, mm^3^/mm^3^), tissue mineral density (TMD, mg HA/cm^3^), and tissue mineral content (TMC, mg HA), defined as BV multiplied by TMD. For statistical analyses, we used SPSS software (Microsoft SPSS for Windows, version 22.0). Assumption of normality was tested with Shapiro‐Wilk test. Dependent on its outcome, within‐subject effects over time were calculated using either a Wilcoxon test or a pairwise comparison of means using a dependent group *t* test. Groups were compared using either a nonparametric Mann–Whitney *U* test or an independent group *t* test. All *p* values were adjusted for multiple testing using Hochberg procedure. Adjusted *p* values <0.05 were considered to indicate significance.

### Biomechanical testing

Immediately after the last μCT scan at day 42, rats were euthanized. Osteotomized femora (*n* = 16) were harvested and fixed in 4% paraformaldehyde for 48 hours. Soft tissue and external fixators were removed. Eventually, bones were washed in tap water for 30 minutes and subsequently transferred into buffer solution for 24 hours before final storage at −80°C. After thawing, epiphyseal bone ends were embedded with methylmethacrylate (Technovit 3400, Heraeus Kulzer, Hanau, Germany) into custom‐made casting containers, mounted into the testing machine (Bose ElectroForce LM1, Bose Corporation, Eden Prairie, MN, USA), and axially preloaded with 0.3 N followed by torsional loading with 0.54°/s crosshead rotation until failure. We analyzed maximum torque at failure and torsional stiffness. Normal distribution was tested using a Shapiro‐Wilk test, and differences between groups were determined using an independent *t* test. All *p* values were adjusted for multiple testing using Hochberg procedure. Adjusted *p* values <0.05 were considered to indicate significance.

### RNA isolation and quantitative real‐time PCR (qPCR)

Callus tissue was harvested at days 3, 7, and 14 post‐operation. Samples were shock‐frozen in liquid nitrogen, pulverized, and dissolved in Trizol (Invitrogen, Carlsbad, CA, USA). Total RNA of each animal was isolated (RNeasy Mini Kit, Qiagen, Valencia, CA, USA) and 1 μg transcribed into cDNA (qScript cDNA SuperMix, Quantabio, Beverly, MA, USA). Measurements were performed in technical triplicates (PerfeCta SYBR Green SuperMix for iQ, Quantabio). Target gene expression was quantified relative to cyclophilin A^1^ using the ΔΔCt method. Primer sequences are given in Supplemental Table S1.

### Sample preparation for arrays

RNA integrity was confirmed (Agilent RNA 6000 Nano Kit; Agilent Technologies, Santa Clara, CA, USA). Per experimental condition, *n* = 4 samples were pooled, 500 ng total RNA labeled (Agilent single‐color Quick‐Amp Labeling Kit), and hybridized on Agilent SurePrint G3 Rat Whole Genome microarrays (8×60K, design‐ID: 028279). Slides were scanned on an Agilent scanner, and feature extraction was performed using Agilent FE software with protocol GE1_1105_Oct12.

### Microarray data analysis

Preprocessing was performed based on the Agi4×44PreProcess package (version 1.18.0), which is implemented in R (http://www.r-project.org) with slight modifications to adapt to the 8×60K format (foreground MeanSignal, background BGMedianSignal). After quantile normalization, gene‐level summarization on log2 scale was annotated to gene symbols based on the grid file 028279_D_F_20131202. For genes represented by multiple probes, average log expression was taken and assigned to the corresponding gene name. Singular value decomposition was performed based on the *svd* function in R. For the heatmaps representing genes involved in different biological processes, expression levels were rescaled to a minimum of zero and a maximum of one. If the mean expression over all samples was below the 25th quantile, genes were excluded to avoid overinterpretation of background noise. Statistical analysis was assessed based on variance calculated for each experimental group (rigid, semi‐rigid, and flexible) over a time course of three measurements (days 3, 7, and 14 post‐operation) as well as for each time point over the three experimental groups. From each analysis, the top 1.5% genes with the highest variance were taken. A total of 285 high‐variance genes were represented within at least two lists. For hierarchical clustering, expression levels were scaled to a mean of zero and a standard deviation of one. Gene ontology (GO) analyses were performed using Ontologizer with settings “Parent‐child union” and “Bonferroni” multiple testing correction. GO terms with adjusted *p* < 0.05 were assessed as significant.

### Second‐harmonic generation (SHG) imaging

Because of high‐molecular noncentrosymmetry and highly organized helical structure, collagen fibrils exhibit endogenous second‐harmonic generation signals.^42^, ^43^ Second harmonic was generated with 100 fs pulse width at 80 MHz and wavelength of 910 nm (Spectra‐Physics Ti:Sapphire laser, Mai Tai HP; Spectra‐Physics, Santa Clara, CA, USA). An internal photomultiplier (Leica SP5 II microscope, Leica Microsystems, Buffalo Grove, IL, USA) was used to detect the SHG signal (450 to 460 nm). Images were recorded using a 25× water immersion objective with numerical aperture of 0.95. Overview images were recorded via image stitching. Collagen fibril orientation was visualized using the freely accessible ImageJ plug‐in FibrilTool^44^ implemented into a macro function which executes orientation analysis within multiple sub‐ROIs of defined size within a gridlike pattern covering the defect site.

## Results

### Histology and μCT analyses

#### Control groups

The 1‐mm osteotomy (positive control) and the untreated 5‐mm defect (negative control) were established by our group for mimicking physiological bone healing and nonunion formation.^37^ The positive control was characterized by woven bone with cartilage islands at the periosteum (periosteal callus), whereas a proliferative tissue matrix filled the periosteal and endosteal defect site at day 7 and day 14. Woven bone separated endosteal sites from medullary canals (endosteal callus). In the negative control, defect sites were filled with fibrous connective tissue and hematoma residues at day 7. Residues of the absorbable collagen sponge were still detectable at day 7 and day 14. Close to osteotomy ends, where surgical cut marks were still visible, there was periosteal woven bone with cartilage tissue (Supplemental Fig. S1).

#### Mechanically regulated BMP2‐induced defect repair

**Figure 2:**
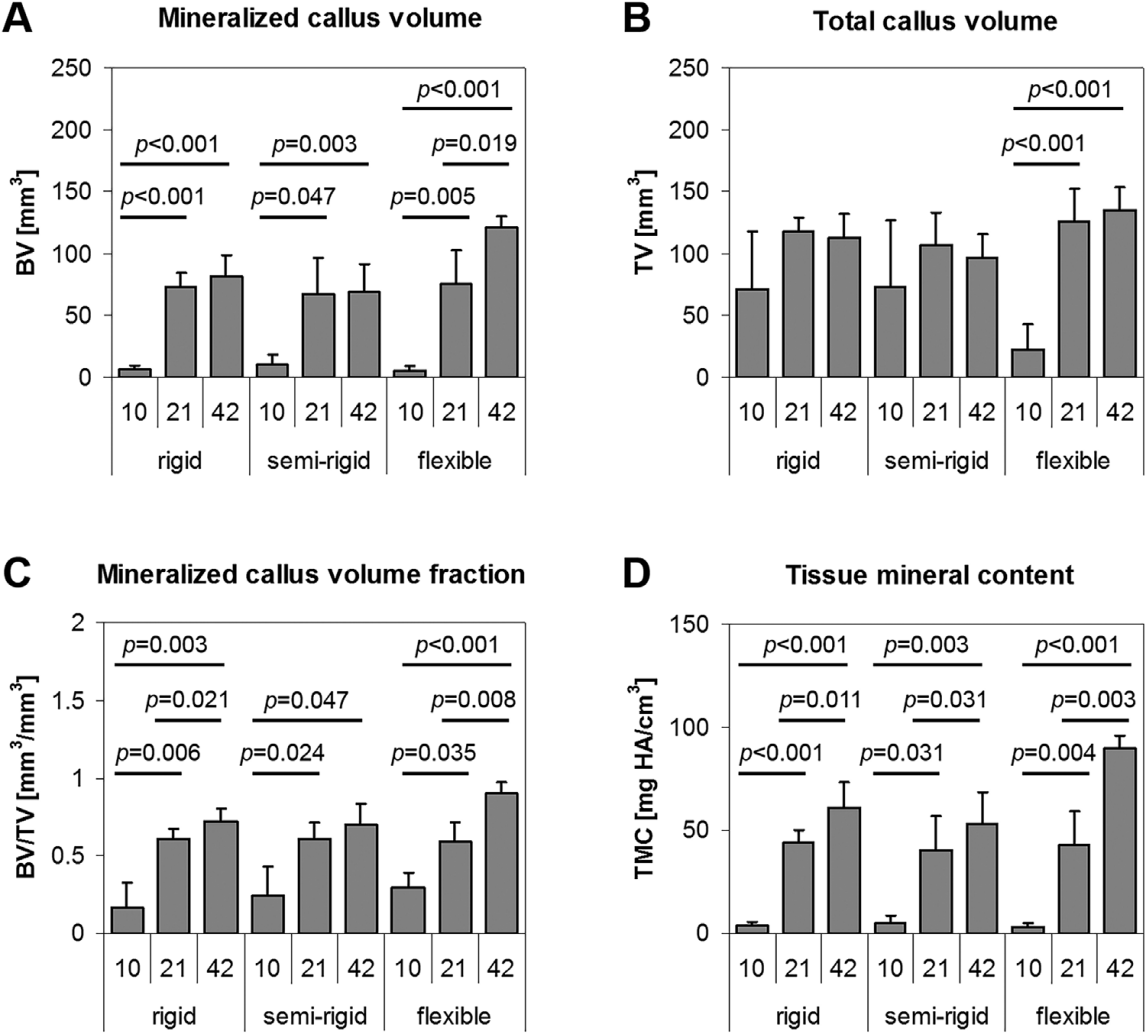
Mineralization of the callus tissue at days 10, 21, and 42 post‐operation. The flexible fixator stability revealed a significant increase of mineralized callus volume (*A*) over time that resulted in significantly more mineral tissue content by day 42 compared with the more rigid groups. In contrast, the rigid and semi‐rigid fixation resulted in slightly reduced total callus volume (*B*) with slight increases in mineralized callus volume fraction (*C*) and tissue mineral content (*D*) by day 42, indicating the onset of fracture remodeling.

At day 7, healing was very similar in all three stiffness groups. Defects were filled with fibrous connective tissue, the collagen sponge, and hematoma residues. Periosteal woven bone was covered with cartilage islands close to the osteotomy ends where surgical cut marks were still visible (Fig. 1 *B*). At day 10, in all stiffness groups, moderate bone formation was detected along the periosteal defect site that was not sufficient for defect bridging. Although statistically not significant, the mineralized callus volume (BV) was largest upon semi‐rigid fixation (Fig. 2 *A*), whereas total callus volume (TV) was lowest upon flexible stabilization (Fig. 2 *B*). Upon rigid and semi‐rigid fixation at day 14, we observed newly formed bone surrounded by cartilage islands at the endosteal defect site. Upon flexible stabilization, there was less woven bone associated with higher numbers of proliferating cells inside the gap and huge cartilage islands at the periosteal site opposite to the fixator (Fig. 1 *B*). By day 21 and day 42, defects were bridged with bone tissue in all groups. Significant differences between the flexible and the more rigid groups occurred at day 42. Total callus volume (TV) was significantly larger upon flexible stabilization compared with semi‐rigid fixation (*p* < 0.03) but not rigid fixation. Upon flexible stabilization, we observed significantly more mineralized callus volume (BV, flexible versus rigid and flexible versus semi‐rigid: *p* < 0.01), tissue mineral content (TMC, flexible versus rigid and flexible versus semi‐rigid: *p* < 0.01), and mineralized callus volume fraction (BV/TV, flexible versus rigid and flexible versus semi‐rigid: *p* < 0.01), indicating a delayed boost of bone formation (Supplemental Tables S2 and S3).

#### Mineralization of callus tissue over time

Flexible stabilization significantly enhanced mineralized callus volume (BV, each *p* < 0.02, Fig. 2 *A*), mineralized callus volume fraction (BV/TV, each *p* < 0.04, Fig. 2 *C*), and tissue mineral content (TMC, each *p* < 0.005, Fig. 2 *D*), whereas tissue mineral density (TMD) significantly increased later between day 21 and day 42 (TMD, *p* < 0.001, Supplemental Table S3). Total callus volume (TV) displayed an accented increase between day 10 and day 21 (*p* < 0.001, Fig. 2 *B*). In contrast, upon rigid and semi‐rigid fixation, the total callus volume (TV) slightly decreased between day 21 and day 42, whereas BV/TV increased significantly over healing time upon rigid fixation (each *p* < 0.03), but only at the earlier time points upon semi‐rigid fixation (day 10 versus day 21: *p* < 0.03). TMC increased significantly in both groups over healing time (rigid: each *p* < 0.02, semi‐rigid: each *p* < 0.04). Differences between the more rigid groups occurred in tissue mineral density (TMD), a marker for bone maturation, which increased significantly over time upon semi‐rigid fixation (day 10 versus day 21: *p* < 0.03, and day 21 versus day 42: *p* < 0.001) but later between day 21 and day 42 upon rigid fixation (*p* < 0.001, Supplemental Table S3).

## Biomechanical assessment of the callus tissue

Differences of the maximum torque at failure of femora harvested 42 days post‐operation were not significant. Rigid fixation resulted in the highest torque with 310.5 ± 44.5 Nmm, followed by semi‐rigid (264.3 ± 66.3 Nmm) and flexible fixation (239.1 ± 96.0 Nmm), suggesting that fixator stiffness modified bone quality despite the same BMP2 treatment. Although statistically not significant, torsional stiffness showed the highest value upon semi‐rigid fixation (43.3 ± 10.3 Nmm/°) followed by rigid fixation (40.0 ± 15.8 Nmm/°) and flexible fixation (38.0 ± 27.8 Nmm/°), suggesting a different progress in bone maturation.

## Candidate gene expression analyzed by qPCR

**Figure 3:**
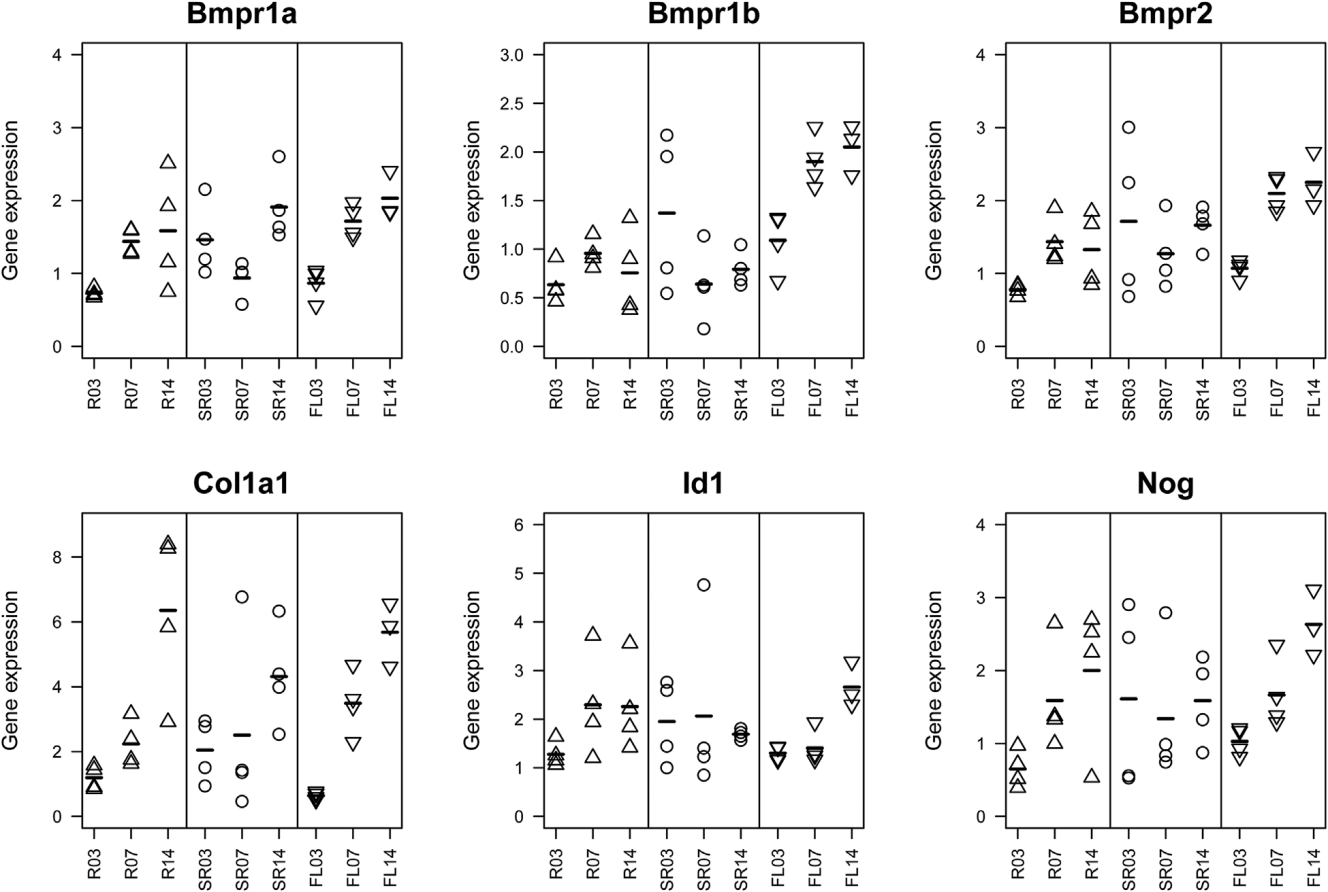
Expression analyses of candidate genes. Genes were selected from a previous in vitro study that identified mechano‐responsive cascades in the BMP signaling path.^(25)^ Expression levels of *n *= 4 animals per group and per time point are displayed (R03 corresponds to rigid group at day 3; SR = semi‐rigid group; FL = flexible group; 07 = day 7; 14 = day 14). Although all animals were treated with the same amount of BMP2, we observed differential regulation of BMP receptor genes (top) and the BMP antagonist *Noggin* (bottom right). *Col1a1* is upregulated over time in all treatment groups. However, in the later time points, we observed increasing variance within groups. Expression pattern of *Id1* shows lower levels at day 3 upon rigid fixation and at days 3 and 7 upon flexible stabilization. For each gene, relative expression is shown. Symbols represent single animals, and bars indicate mean expression at the indicated time points.

We wondered whether differential gene expression accompanies the modulation of healing courses by fixator stiffness (Fig. 3). Candidate genes were selected from a previous in vitro study that demonstrated differential regulation of BMP target genes such as *Id1* and *Nog* by BMP2 and mechanical loading where *Id1* and *Nog* showed induction by BMP2 treatment that was boosted by mechanical loading.^(25)^ In our analyses, *Id1* showed lowest expression upon rigid fixation at day 3, upon flexible fixation at days 3 and 7, and highest mean expression upon flexible fixation at day 14. Mean expression of *Noggin* and *Bmpr1a* increased over time upon rigid and flexible fixation, whereas it remained on moderate levels over all time points upon semi‐rigid fixation. Mean expression of *Col1a1* increased over time in all stiffness groups. Interestingly, it was lowest upon flexible stabilization at day 3, whereas it was higher in this group at day 7 in comparison to the other two groups and highest upon rigid fixation at day 14. Mean expression of *Bmpr1b* increased upon flexible stabilization, reaching highest expression at day 14. Taken together, we observed differences in gene expression dependent on the fixator stiffness that required further exploration.

## Microarray‐based characterization of the impact of fixator stiffness on BMP2‐induced defect healing

Observing high variance of expression levels as determined by qPCR that were not dependent on distinct candidate genes, time points, or stiffness groups, we estimated that we would need even more than 4 animals per experimental group and time point to obtain statistical significance based on single animals. To avoid large animal numbers, we decided to conduct a descriptive exploratory analysis based on pooled RNA samples, which, of course, results in a lack of statistical power. First, expression data were analyzed using singular value decomposition (Supplemental Figs. S2–S6). The top four components explained more than 80% of variance (Supplemental Fig. S2). Differential expression over time mainly contributed to variance in gene expression. Genes contributing to component 3, which explains about 14% of variance, reflect the three different fixator stiffness groups at day 7 (Supplemental Fig. S5). Top 100 genes contributing to each of the four components were subjected to GO analyses. Prominent terms were related to immune system, cytokine activity and production, ECM and ossification, as well as contractile fiber, cellular contraction, and actin‐filament‐based processes.

**Figure 4:**
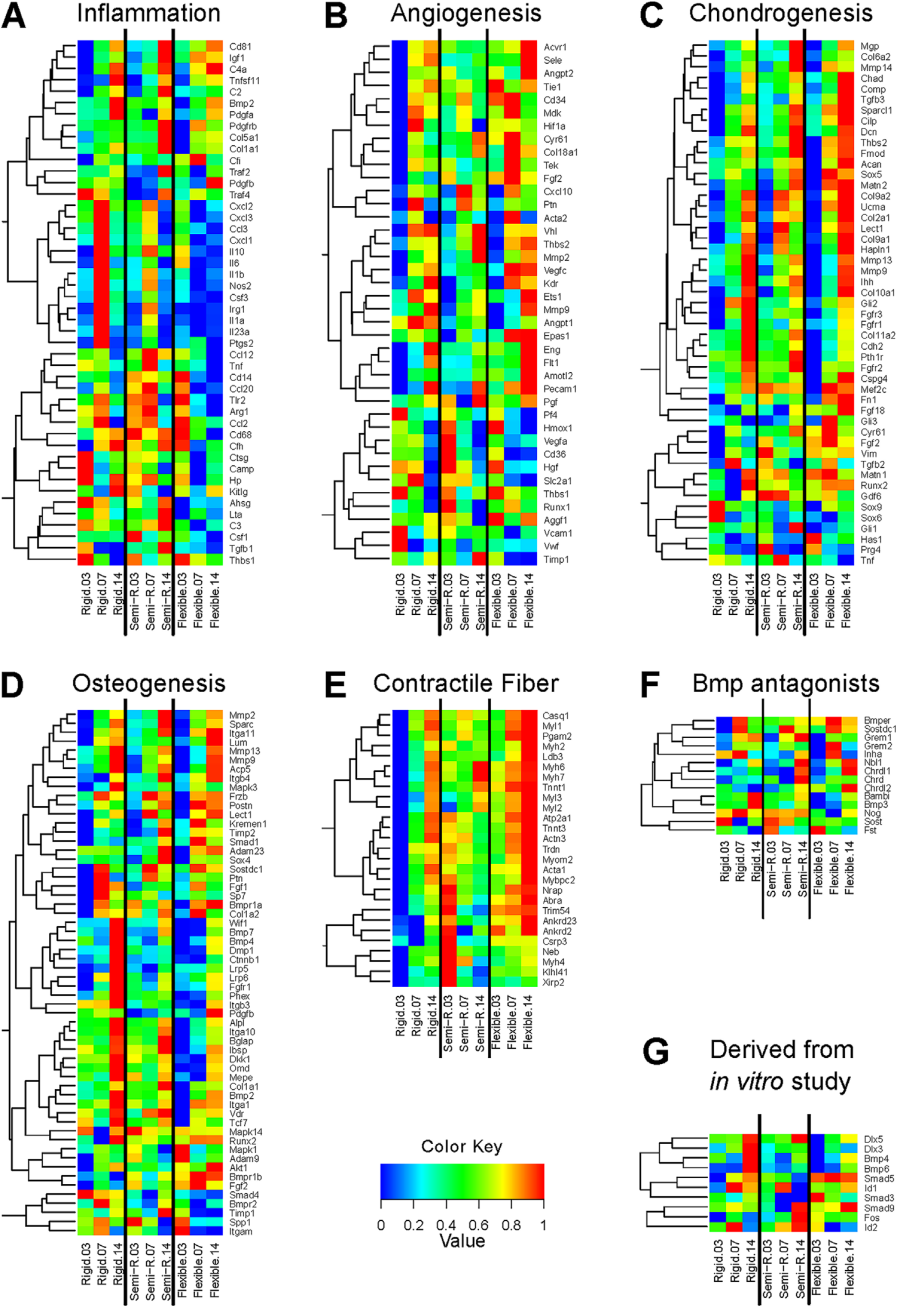
Genes involved in biological processes that are known to play critical roles during fracture healing are differentially expressed dependent on fixator stiffness. Genes were selected that are involved in (*A*) inflammation, (*B*) angiogenesis, (*C*) chondrogenesis, and (*D*) osteogenesis. (*E*) Expression of high‐variance genes annotated to “contractile fiber” illustrates a strong interaction of BMP stimulation with fixator stiffness. (*F*) Expression levels of known BMP antagonists. (*G*) Genes that were analyzed earlier in vitro by Kopf and colleagues^25^ and not included in *A–F*. (*A–G*) Vectors of the expression levels of each gene were rescaled to a minimum of zero and a maximum of one.

Next, we selected marker genes for different stages of bone healing. Inflammation (Fig. 4 *A*) is the initial response to tissue damage. Pro‐inflammatory cytokines such as *Il6* and *Il1b* as well as chemokines such as *Cxcl1*, *Cxcl2*, *Cxcl3*, and *Ccl3* peaked at day 7 upon semi‐rigid and rigid fixation, whereas this peak was less pronounced upon semi‐rigid fixation. Upon flexible stabilization, these genes displayed moderate expression at day 3 and downregulation later on. The anti‐inflammatory factor *Il10* was highly expressed at day 3 upon flexible stabilization and subsequently downregulated. Upon semi‐rigid fixation, *Il10* was continuously expressed but peaked at day 7. Upon rigid fixation, *Il10* was strongly upregulated at day 7 and still expressed at day 14. The inflammatory factors TNF‐α (*Tnf*), RANKL (*Tnfsf11*), and M‐CSF (*Csf1*) are involved in endochondral ossification, ie, replacement of calcified cartilage by woven bone. *Tnf* was expressed at day 3 in all groups but had the strongest expression in the semi‐rigid group at day 7 and was downregulated in all groups by day 14. *Tnfsf11* was upregulated over time in all groups. Expression was only modest at day 7 upon rigid fixation but reached highest level in this group at day 14. *Csf1* was continuously expressed at medium level upon rigid fixation, showed peak expression upon semi‐rigid fixation at day 3 and lowest expression upon flexible stabilization at day 14. Taken together, the initial step of the healing cascade is influenced by fixator stiffness.

VEGF and angiopoietin signaling regulate blood vessel formation. Several genes related to angiogenesis (Fig. 4 *B*) displayed lowest expression upon rigid fixation at day 3. *Vegfa* displayed highest expression at day 3 and lowest expression at day 14 upon semi‐rigid fixation. At day 7, there was a peak upon rigid fixation but downregulation upon flexible stabilization. In contrast, *Vegfc* was upregulated in all groups with most prominent upregulation upon flexible stabilization. Angiopoietin1 (*Angpt1*) was higher expressed upon rigid fixation than upon semi‐rigid fixation with a peak at day 7, whereas expression was much lower upon flexible stabilization. In contrast, *Angpt2* revealed the highest expression upon flexible stabilization at day 14 and the lowest expression at day 3 upon rigid fixation. With bone damage, blood vessel injuries cause hypoxia at the defect site. The hypoxia activated factor HIF‐1α (*Hif1a*) was moderately highly expressed upon flexible and semi‐rigid fixation but downregulated at day 14 upon semi‐rigid fixation. In contrast, *Hif1a* showed a prominent peak at day 7 upon rigid fixation. In line with this observation, Glut‐1 (*Slc2a1*), a HIF‐1α target gene, showed highest expression upon rigid fixation at day 7. The pro‐angiogenic and anti‐inflammatory factor HMOX (*Hmox1*) showed peak expression at day 3 in all groups where expression was highest upon flexible stabilization and lowest upon rigid fixation. Overall, fixator stiffness influenced expression pattern of genes related to angiogenesis.

Expression of the majority of genes related to chondrogenesis (Fig. 4 *C*) increases over time. Several genes showed lowest expression at day 3 and highest expression at day 14 upon flexible stabilization, which is in line with our observation that cartilaginous matrix formation is delayed and more strongly increasing later on (cf, large cartilaginous islands at day 14, Fig. 1 *B*). However, some chondrogenic marker genes such as *Chad* and *Comp* are lower expressed upon more rigid fixation. Endochondral ossification is characterized by bone formation via calcified cartilaginous matrix. *Col2a1*, *Col9a1*, and *Col9a2* encoding collagen components of cartilage, as well as *Lect1* (chondromodulin), which is suggested to promote chondrocyte growth and to inhibit angiogenesis, displayed lower expression at day 14 than at day 7 upon semi‐rigid fixation, whereas expression levels were still rising between day 7 and day 14 in the other groups. This might indicate an earlier preponderance of mineralization upon semi‐rigid fixation.

Most of the genes related to osteogenesis (Fig. 4 *D*), such as *Dkk1*, *Dmp1*, *Mepe*, *Phex*, *Lrp5*, and *Lrp6*, displayed highest expression levels upon rigid fixation at day 14. However, *Col1a1* and marker genes for tissue mineralization (*Bglap*, *Ibsp*, *Mepe*) showed highest expression levels upon semi‐rigid fixation at day 14. Expression of *Timp1* and *Timp2* encoding inhibitors of matrix metalloproteinases also peaked here, whereas expression levels of the matrix metalloproteinases *Mmp9* and *Mmp13* were lower at this time point in comparison to the rigid and flexible group. This might indicate a lower demand on matrix remodeling upon semi‐rigid fixation. Upon flexible stabilization, delayed upregulation of the majority of genes related to osteogenesis is in line with delayed and augmented mineralization as observed by histology and μCT.

Expression pattern of BMP antagonists (Fig. 4 *F*) did not follow a distinct rule. We note, that peak expression was not observed at day 3 indicating that BMP2 stimulation was not impaired by endogenous upregulation of BMP antagonists.

Genes derived from a study by Kopf and colleagues focusing on the interaction of mechanical loading and BMP signaling in vitro,^25^ including *Runx2* (Fig. 4 *C*), *Bmp2*, *Bmpr1a*, *Bmpr1b*, *Bmpr2*, *Bmp7* (Fig. 4 *D*), *Nog* (Fig. 4 *F*), *Dlx5*, *Dlx3*, *Bmp4*, *Bmp6*, *Smad5*, *Id1*, *Smad3*, *Smad9*, *Fos*, and *Id2* (Fig. 4 *G*), revealed differences in expression profile dependent on fixator stiffness. This suggests that the mechano‐responsivity of their expression is also detectable in vivo. Comparison of candidate gene expression based on qPCR (Fig. 3) and corresponding expression levels as determined by microarrays (Fig. 4) revealed minor differences (Supplemental Fig. S7).

**Figure 5:**
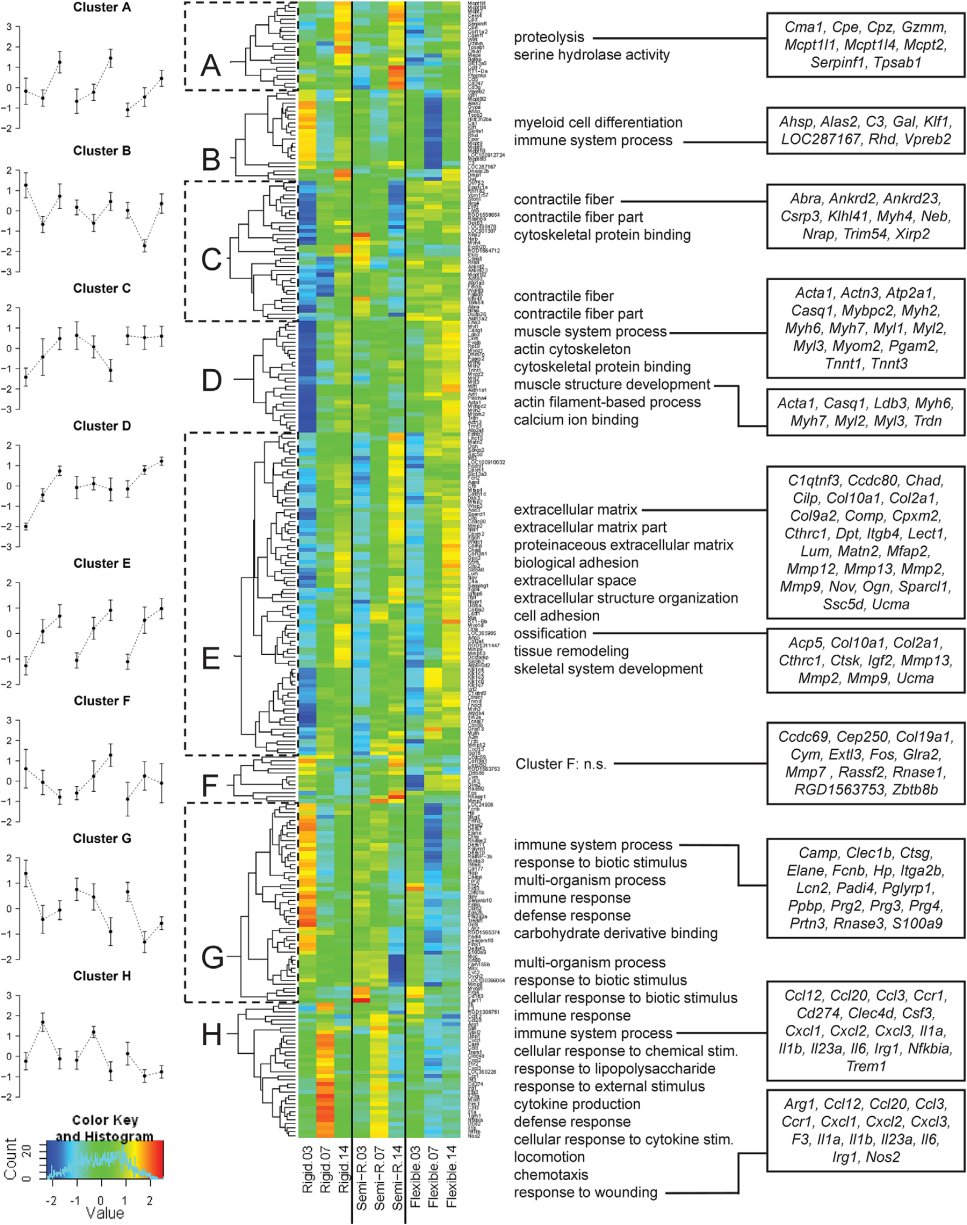
The interaction of BMP2 treatment and fixator stiffness alters biological and cellular processes related to ECM formation, contractile fiber organization, response to wounding, immune system, and ossification. Expression levels of each gene were scaled to a mean of 0 and standard deviation of 1. Based on these vectors, hierarchical clustering was performed and eight clusters were identified by visual inspection (*A–H*). Line plots (left) display the mean expression and standard deviation of all genes per cluster. GO analyses (middle) were performed on each cluster, and terms with an adjusted *p* < 0.05 are shown. For clarity, more unspecific GO terms with population size >2500 were excluded. Genes, annotated to prominent GO terms, are shown on the right side.

For unsupervised exploration, filtering of candidate genes was assessed based on variance between experimental groups and time points. From each analysis, the top 1.5% genes with the highest variance were taken, which resulted in a total of 285 genes. Eight major clusters were identified by hierarchical clustering (Fig. 5). Cluster A includes the genes *Bglap* and *Mepe*, which play a key role in matrix mineralization, and genes that are mainly related to “proteolysis” and “serine hydrolase activity.” These genes showed increasing expression levels in all stiffness groups with highest expression levels at day 14 in the semi‐rigid group. The 23 genes in cluster B display an intermittent drop of expression at day 7. Interestingly, the overall expression pattern is shifted dependent on fixator stiffness with higher levels upon rigid fixation and lower levels upon flexible stabilization. The majority of these genes are associated with immunological processes. Additionally, cluster B included *Dmp1*, which is a negative regulator of osteoblast proliferation and involved in ossification. Cluster C and D show expression patterns that differ between stiffness groups. All of these genes show increasing expression levels over time upon rigid fixation. The 35 genes in Cluster C show decreasing expression levels over time upon semi‐rigid fixation and high expression over all time points upon flexible stabilization. About half of these genes as well as 15/28 genes in cluster D are annotated to “contractile fiber” including numerous genes encoding myosin heavy and light chains. Cluster E is the largest cluster comprising 81 genes. These genes display increasing expression over time in all stiffness groups with a more prominent increase between day 3 and day 7. Products of 53 of these genes are localized in the extracellular region, and several of them such as diverse collagens and matrix‐metalloproteinases play a well‐known role in connective tissue and bone biology. Cluster F (12 genes) includes *Fos* and *Mmp7*, but GO analysis revealed no significant term. In cluster G, 17/50 genes are annotated to “immune system process.” These genes show highest expression levels at day 3 and at this time point highest expression upon rigid fixation. Genes such as *Camp*, *Ctsg*, *Elane*, *Hp*, *Itga2b*, *Lcn2*, *Padi4*, *Ppbp*, *Prtn3*, and *S100a9* are highly expressed at sites of connective tissue remodeling and inflammation. Cluster H comprises 34 genes that display peak expression levels at day 7 upon rigid and semi‐rigid fixation. Upon flexible stabilization, there is moderate expression at day 3 comparable with expression levels in the other two groups, but then expression levels decrease. Numerous genes in cluster H are expressed by macrophages such as *Arg1*, *Ccl20*, *Ccl3*, *Ccr1*, *Cd274*, *Clec4d*, *Csf3*, *Cxcl1*, *Cxcl2*, *Cxcl3*, *F3*, *Il1a*, *Il1b*, *Il1r2*, *Il23a*, *Il6*, *Irg1*, *Nos2*, *Trem1*, or regulate macrophage activation such as *Nfkbia* and *Slpi*. Similar to cluster B, the overall expression pattern is shifted dependent on fixator stiffness with higher levels in the rigid group. Differential expression of the 285 high‐variance genes in the control groups is shown in Supplemental Fig. S8. Taken together, our microarray analyses confirmed that fixator stiffness modulates expression of genes associated with a variety of biological processes including a) immunological processes, b) ECM reorganization, formation and mineralization, and unexpectedly c) cellular contraction.

## Analysis of ECM alignment

**Figure 6:**
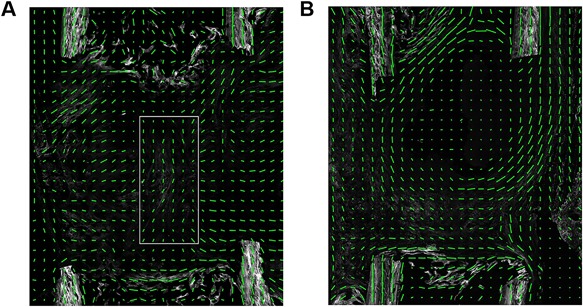
Matrix alignment. (*A*) Flexible group at day 14. Fiber orientation in the middle of the defect (white box) runs in parallel to the bone axis (cf, Supplemental Fig. S9*A*). (*B*) Semi‐rigid group at day 14. Fiber orientation at the margins of the fracture site runs in parallel to the bone axis and perpendicular fibers seal the medullary canal (cf, Supplemental Fig. S9*B*). (*A*, *B*) A green line in each sub‐ROI indicates the direction of primary fibril orientation, and the length of the line represents the degree of anisotropy.

As shown in Fig. 4 *E*, lowest expression levels of selected genes related to “contractile fiber” were detected upon rigid fixation in particular at day 3, whereas expression is higher upon semi‐rigid fixation and highest upon flexible stabilization at day 14 in the majority of genes. *Myh6* encodes a myosin heavy chain α isoform (MHC‐α), which is predominantly expressed in the cardiac atria, whereas *Myh7* encodes a myosin heavy chain β isoform (MHC‐β) expressed in the cardiac ventricles and skeletal muscles (type I fibers). Both are proteins of myosin II, which is not only responsible for striated muscle contraction but also, together with Alpha actin 1 (*Acta1*), involved in cell motility. Because fiber alignment within the ECM is associated with cellular migration, we used SHG microscopy to visualize fiber orientation within nonmineralized defect sites at day 14. Upon flexible stabilization, fiber orientation resembled the shape of a sandglass (Fig. 6 *A*, Supplemental Fig. S9*A*), whereas upon semi‐rigid fixation, fiber orientation was reminiscent of a crate or bowl‐shaped shelter (Fig. 6 *B*, Supplemental Fig. S9*B*). Thus, fixator stiffness modulated matrix alignment.

## Discussion

Although widely used, the detailed mechanism by which BMP2 allows the orchestration of mature bone formation in BMP2‐induced bone defect regeneration remains unknown. Recently, work of others and us has illustrated the mechano‐sensitivity of BMP2 treatment. In a rat critical‐sized defect we previously demonstrated increased mineralized tissue formation and remodeling rate after combined treatment (BMP2 and mechanical loading) in comparison to BMP2 only treatment.^32^ In the present study, we reduced the BMP2 dosage by a factor of 10, performed physiological loading by weight bearing, and used tissue and molecular analyses to characterize the interplay of mechanical and biological stimulation in defect repair on gene expression level. This work has direct practical implications on how fractures and defects should be stabilized to maximize the effect of BMP2 proteins.

The distinct lower dosage of 5 μg BMP2 induced bone healing in our critical‐sized defects stabilized with external fixation. The semi‐rigid stabilized defect represented the best healing outcome with an earlier formation and maturation of mineralized tissue. The same external fixator set (RISystem, Davos, Switzerland; 100%, 40%, and 10%) and gap size (5 mm), with 5.5 μg BMP2, has been tested by Glatt and colleagues.^33^, ^39^ They included an additional fixation stiffness of 70% that revealed best defect healing in male rats, whereas we had the best healing outcome within the 40% group in female rats. A possible reason for this discrepancy could be the rats’ sex because bony defects are known to heal better in male Sprague‐Dawley rats than in females.^37^ Possibly, optimal fixator stiffness is somewhere between 40% and 70%. A limitation of the animal model is the material of the fixator bars (polyethylethylketone) because we observed bite and gnaw marks. The local legal representative approved application of protective collars to prevent further actions.

The gene expression analyses included in our study illustrate that the mechano‐sensitivity of BMP2 signaling affected gene expression as early as 3 days after surgery. Numerous genes related to chondrogenesis and osteogenesis displayed lowest expression levels upon flexible stabilization, reflecting differential response to mechanical environment. In contrast, genes related to inflammatory response and immune system processes as well as genes known to be highly expressed by macrophages were higher expressed at day 3 and elevated levels were even more prominent at day 7 upon rigid fixation. The pronounced expression of genes related to ECM remodeling as well as genes highly expressed by macrophages upon rigid fixation might indicate a higher degree of softening of the ECM to allow for sufficient strain to obtain mechanical guidance. In line with this, genes related to cellular contractility are much lower expressed at these time points upon rigid fixation.

Cellular contraction is not restricted to cellular migration and organization of the ECM but is also a pivotal process in ECM stiffening. In previous studies, we observed differential expression of genes involved in cellular contraction in large and small animal models of bone defect healing.^31^, ^45^ Based on the results of the exploratory analyses described here, we performed a targeted in vitro experiment. Using a custom‐made mechano‐bioreactor system,^38^ we confirmed that cyclic monoaxial compression significantly stimulated scaffold volume contraction and scaffold stiffness as determined by Young's modulus (Supplemental Fig. S10). To elucidate gene regulatory mechanisms mediating cellular and ECM contraction in the context of mechanical loading in more detail, further research will be necessary.

Contractility is involved in cell migration, cell constriction, cell‐cell intercalation, and junction modeling.^46^ Additionally, contractility can facilitate and accelerate cellular alignment to traction forces and static strain. Interestingly, cell alignment does not necessarily follow preexisting matrix fiber orientation,^47^ but cells can use contractility to align ECM fibers.^16^ Using SHG imaging on nonmineralized sections, we were able to visualize a sandglass‐like fiber alignment in the defect gap along the bone axis upon flexible stabilization. We speculate that the cage‐like fiber orientation upon semi‐rigid fixation might facilitate the establishment of tensile forces and ECM stiffening inside and thus the onset of mineralization.

Previous studies demonstrated a prolonged cartilage phase and larger amounts of bone tissue associated with a larger bony callus in the context of unstable fixation.^48^, ^49^ Here we showed that this association can still be observed in the context of BMP2 treatment. Upon more rigid fixation, we observed an earlier onset of osteogenesis compared with flexible stabilization, indicating a different micromechanical environment that caused reduced or delayed osteogenesis despite BMP2 treatment. The short biological half‐life of administered rhBMP2 limits its action on a rather short period of time. For example, osteoblast differentiation is accompanied with upregulation of miR‐497‐195 cluster microRNAs, which dramatically downregulate BMP2 responsiveness.^50^ Thus, the major effect of BMP2 administration is to enforce osteogenic lineage commitment. Zouani and colleagues^51^ demonstrated that osteogenic induction of mesenchymal stem cells is dependent on high substrate rigidity. However, when BMP2 was applied, osteogenic lineage commitment also occurred on softer matrices. This effect was abolished when cells were grafted on even softer gels. Applied to our critical‐sized defects, higher interfragmentary movement due to flexible stabilization may lead to ECM disruption, which impairs cell adhesion, spreading, and rigidity acting on stem cells, thus resulting in a predominance of cartilage lineage commitment despite BMP2 treatment.

Gene expression analyses are a powerful tool to detect predominant biological processes. Using this approach, we showed differences in healing as early as 3 days after surgery. Upon flexible stabilization, cells predominantly commit to the cartilage lineage. Fiber alignment is organized in a sandglass‐like structure surrounded by large cartilage islands at the periosteal sites. Upon rigid fixation, there is a higher degree of ECM remodeling and macrophage activity. Cellular contractility and, as a consequence of this, cellular alignment to tensile forces are affected. Upon semi‐rigid fixation, fiber alignment displays a cage‐like structure, which might facilitate cellular alignment to tensile forces, cellular cluster formation, and thus the onset of mineralization inside. Based on our results obtained by SHG analyses, we assume that there is indeed an interaction between contractile fiber gene expression, ECM orientation, and tissue contraction. Further studies are needed to investigate these processes both in vitro and in the context of fracture healing in more detail.

## Disclosures

All authors state that they have no conflicts of interest.

## Supporting information

Additional supporting information may be found in the online version of this article.

Supporting Data S1.
