# Supplementary material for: The Interaction of BMP2‐Induced Defect Healing in Rat and Fixator Stiffness Modulates Matrix Alignment and Contraction
Source: JBMR Plus. 2018 Apr 17;2(3):174–86. doi: 10.1002/jbm4.10031 (PMC6124159; doi:10.1002/jbm4.10031)
Supplement: Supplementary file 1 — Supporting Data S1. [file JBM4-2-174-s001.pdf]

Supplementary Information

Title:

The interaction of BMP2-induced defect healing in rat and fixator stiffness modulates matrix alignment and contraction

Authors:

Carolin Schwarz[1,2,#], Claus-Eric Ott[3,4,#], Dag Wulsten[1], Erik Brauer[1], Sophie Schreivogel[1], Ansgar Petersen[1,2], Kerstin Hassanein[1], Linda Roewer[1], Tanja Schmidt[1,2], Bettina Willie[5], Georg N. Duda[1,2,*]

Affiliations:

1.) Julius Wolff Institute and Center for Musculoskeletal Surgery, Charité – Universitätsmedizin Berlin, corporate member of Freie Universität Berlin, Humboldt-Universität zu Berlin, and Berlin Institute of Health, Berlin, Germany.

2.) Berlin-Brandenburg Center for Regenerative Therapies (BCRT), Charité – Universitätsmedizin Berlin, corporate member of Freie Universität Berlin, Humboldt-Universität zu Berlin, and Berlin Institute of Health, Berlin, Germany.

3.) Institute for Medical Genetics and Human Genetics, Charité – Universitätsmedizin Berlin, corporate member of Freie Universität Berlin, Humboldt-Universität zu Berlin, and Berlin Institute of Health, Berlin, Germany.

4.) Research Group Development and Disease, Max Planck Institute for Molecular Genetics, Berlin, Germany.

5.) Research Center, Shriners Hospitals for Children-Canada, Department of Pediatric Surgery, McGill University, Montreal, Canada.

\# shared first authorship

Corresponding author:

Prof. Dr. Georg N. Duda, Julius Wolff Institute and Center for Musculoskeletal Surgery, Berlin-Brandenburg Center for Regenerative Therapies, Charité – Universitätsmedizin Berlin, Augustenburger Platz 1, Berlin 13353, Germany

Email: georg.duda@charite.de

Contents

Table S1. Sequences of Primers used for qPCR
Table S2. Quantitative *in vivo* MicroCT data
Table S3. Statistical Analyses of Quantitative *in vivo* MicroCT data

Figure S1. Representative histology of the control groups
Figure S2. Singular value decomposition (SVD) of gene expression data
Figure S3. SVD – Component 1
Figure S4. SVD – Component 2
Figure S5. SVD – Component 3
Figure S6. SVD – Component 4
Figure S7. Expression of candidate genes analyzed by qPCR on microarrays
Figure S8. Expression pattern of the 285 high-variance genes in control groups
Figure S9. Second-harmonic generation (SHG) microscopy
Figure S10. Bioreactor-based analysis of load-induced ECM contraction

Supplementary methods

Table S1: Sequences of Primers used for qPCR.

Gene	*Forward*	*Reverse*
Id1	ACTCTGAGTCTGAAGTCGCG	CGGTAGTGTCTTTCCCCGG
Nog	AAGAAGCTGAGGAGGAAGTTACAG	GCACAGACTTGGATGGCTTAC
Col1a1	GCAACAGTCGATTCACCTACAG	TGGGATGGAGGGAGTTTACA
Bmpr1a	GGAGGAATCGTGGAGGAATA	TGTGAGTCTGGATGCTGGATTA
Bmpr1b	GGAGATGTGTTTCTGGAGGTATAG	GCCCAGCACTCTGTCATAAG
Bmpr2	CCAGAAGCCTGGAAAGAAAATAG	GAGGAAGAGGAATAATCTGGGTAAG
Ppia (ENDO)	GCACTGGTGGCAAGTCCATCT	TGCTCATGCCTTCTTTCACCTTC

Table S2: Quantitative *in vivo* MicroCT data.

Day / Group	Rigid	Semi-rigid	Flexible
**Mineralized callus volume (BV) [mm³]**			
10	5,74 ± 3,05	10,26 ± 7,89	5,21 ± 3,47
21	72,70 ± 12,11	67,00 ± 29,37	75,39 ± 26,87
42	81,34 ± 16,78	69,15 ± 22,45	120,74 ± 9,78
**Total callus volume (TV) [mm³]**			
10	71,04 ± 46,91	72,81 ± 54,33	22,66 ± 19,52
21	118,07 ± 10,66	107,09 ± 26,08	126,15 ± 26,53
42	113,25 ± 18,71	96,85 ± 18,92	135,36 ± 18,07
**Mineralized callus volume fraction (BV/TV) [mm³/mm³]**			
10	0,16 ± 0,16	0,24 ± 0,19	0,29 ± 0,10
21	0,61 ± 0,06	0,61 ± 0,10	0,59 ± 0,12
42	0,72 ± 0,08	0,70 ± 0,13	0,90 ± 0,07
**Tissue mineral content (TMC) [mg HA/cm³]**			
10	3,39 ± 1,97	5,07 ± 3,72	3,05 ± 1,75
21	43,60 ± 6,26	40,04 ± 16,91	42,91 ± 16,24
42	61,11 ± 11,84	53,01 ± 15,06	89,56 ± 6,09
**Tissue mineral density (TMD) [mg HA/cm³]**			
10	587,09 ± 158,18	515,44 ± 36,28	628,26 ± 110,28
21	601,76 ± 27,93	602,46 ± 39,65	564,60 ± 26,26
42	753,79 ± 28,49	777,42 ± 47,48	743,94 ± 55,13

Table S3: Statistical Analyses of Quantitative *in vivo* MicroCT data.

a) Between-group comparisons, BV

Outcome measure	Stiffness	Day	Shapiro-Wilks (normality)	equal variance ttest	Mann-Whitney-U	final adj *p*-value Hochberg
BV	Rigid<br>Semi-rigid	10	0.302<br>0.854	**0.261**	0.537	0.392
BV	Rigid<br>Flexible	10	0.302<br>0.466	**0.803**	0.690	0.803
BV	Semi-rigid<br>Flexible	10	0.854<br>0.466	**0.220**	0.329	0.392
BV	Rigid<br>Semi-rigid	21	0.286<br>0.012	0.696	**0.329**	0.843
BV	Rigid<br>Flexible	21	0.286<br>0.682	**0.843**	0.999	0.843
BV	Semi-rigid<br>Flexible	21	0.012<br>0.682	0.636	**0.662**	0.843
BV	Rigid<br>Semi-rigid	42	0.751<br>0.762	**0.343**	0.429	0.343
BV	Rigid<br>Flexible	42	0.751<br>0.891	**0.002**	0.008	0.003
BV	Semi-rigid<br>Flexible	42	0.762<br>0.891	**0.001**	0.004	0.003

b) Between-group comparisons, TV

Outcome measure	Stiffness	Day	Shapiro-Wilks (normality)	equal variance ttest	Mann-Whitney-U	final adj *p*-value Hochberg
TV	Rigid<br>Semi-rigid	10	0.719<br>0.347	**0.956**	0.999	0.956
TV	Rigid<br>Flexible	10	0.719<br>0.824	**0.066**	0.151	0.125
TV	Semi-rigid<br>Flexible	10	0.347<br>0.824	**0.083**	0.177	0.125
TV	Rigid<br>Semi-rigid	21	0.254<br>0.002	0.404	**0.126**	0.371
TV	Rigid<br>Flexible	21	0.254<br>0.993	**0.545**	0.548	0.545
TV	Semi-rigid<br>Flexible	21	0.002<br>0.993	0.262	**0.247**	0.371
TV	Rigid<br>Semi-rigid	42	0.318<br>0.102	**0.184**	0.126	0.184
TV	Rigid<br>Flexible	42	0.318<br>0.521	**0.094**	0.151	0.141
TV	Semi-rigid<br>Flexible	42	0.102<br>0.521	**0.008**	0.030	0.024

c) Between-group comparisons, BV/TV

Outcome measure	Stiffness	Day	Shapiro-Wilks (normality)	equal variance ttest	Mann-Whitney-U	final adj *p*-value Hochberg
BV/TV	Rigid<br>Semi-rigid	10	0.15<br>0.178	**0.480**	0.329	0.572
BV/TV	Rigid<br>Flexible	10	0.15<br>0.978	**0.138**	0.151	0.414
BV/TV	Semi-rigid<br>Flexible	10	0.178<br>0.978	**0.572**	0.329	0.572
BV/TV	Rigid<br>Semi-rigid	21	0.871<br>0.487	**0.909**	0.999	0.909
BV/TV	Rigid<br>Flexible	21	0.871<br>0.144	**0.673**	0.999	0.909
BV/TV	Semi-rigid<br>Flexible	21	0.487<br>0.144	**0.767**	0.931	0.909
BV/TV	Rigid<br>Semi-rigid	42	0.121<br>0.046	0.838	**0.931**	0.931
BV/TV	Rigid<br>Flexible	42	0.121<br>0.181	**0.005**	0.008	0.008
BV/TV	Semi-rigid<br>Flexible	42	0.046<br>0.181	0.015	**0.004**	0.008

d) Between-group comparisons, TMD

Outcome measure	Stiffness	Day	Shapiro-Wilks (normality)	equal variance ttest	Mann-Whitney-U	final adj *p*-value Hochberg
TMD	Rigid<br>Semi-rigid	10	0.006<br>0.119	0.305	**0.429**	0.429
TMD	Rigid<br>Flexible	10	0.006<br>0.401	0.646	**0.310**	0.429
TMD	Semi-rigid<br>Flexible	10	0.119<br>0.401	**0.041**	0.052	0.123
TMD	Rigid<br>Semi-rigid	21	0.777<br>0.26	**0.975**	0.931	0.975
TMD	Rigid<br>Flexible	21	0.777<br>0.239	**0.062**	0.095	0.153
TMD	Semi-rigid<br>Flexible	21	0.26<br>0.239	**0.102**	0.247	0.153
TMD	Rigid<br>Semi-rigid	42	0.866<br>0.257	**0.357**	0.429	0.536
TMD	Rigid<br>Flexible	42	0.866<br>0.972	**0.732**	0.841	0.732
TMD	Semi-rigid<br>Flexible	42	0.257<br>0.972	**0.307**	0.429	0.536

e) Between-group comparisons, TMC

Outcome measure	Stiffness	Day	Shapiro-Wilks (normality)	equal variance ttest	Mann-Whitney-U	final adj *p*-value Hochberg
TMC	Rigid<br>Semi-rigid	10	0.363<br>0.92	**0.391**	0.537	0.587
TMC	Rigid<br>Flexible	10	0.363<br>0.463	**0.775**	0.841	0.775
TMC	Semi-rigid<br>Flexible	10	0.92<br>0.463	**0.296**	0.429	0.587
TMC	Rigid<br>Semi-rigid	21	0.491<br>0.002	0.671	**0.082**	0.246
TMC	Rigid<br>Flexible	21	0.491<br>0.48	**0.935**	0.999	0.935
TMC	Semi-rigid<br>Flexible	21	0.002<br>0.48	0.782	**0.931**	0.935
TMC	Rigid<br>Semi-rigid	42	0.875<br>0.716	**0.355**	0.537	0.355
TMC	Rigid<br>Flexible	42	0.875<br>0.81	**0.001**	0.008	0.002
TMC	Semi-rigid<br>Flexible	42	0.716<br>0.81	**0.001**	0.004	0.002

f) Time-series comparisons, BV

Outcome measure	Day	Stiffness	Shapiro-Wilks (normality)	equal variance ttest	Wilcoxon	final adj *p*-value Hochberg
BV	10<br>21	Rigid	0.302<br>0.286	**0.000**	0,063	0.000
BV	10<br>42	Rigid	0.302<br>0.751	**0.000**	0,063	0.000
BV	21<br>42	Rigid	0.286<br>0.751	**0.244**	0,313	0.244
BV	10<br>21	Semi-rigid	0.854<br>0.012	0.003	**0.031**	0.047
BV	10<br>42	Semi-rigid	0.854<br>0.762	**0.001**	0.031	0.003
BV	21<br>42	Semi-rigid	0.012<br>0.762	0.756	**0.844**	0.844
BV	10<br>21	Flexible	0.466<br>0.682	**0.003**	0,063	0.005
BV	10<br>42	Flexible	0.466<br>0.891	**0.000**	0,063	0.000
BV	21<br>42	Flexible	0.682<br>0.891	**0.019**	0,063	0.019

g) Time-series comparisons, TV

Outcome measure	Day	Stiffness	Shapiro-Wilks (normality)	equal variance ttest	Wilcoxon	final adj *p*-value Hochberg
TV	10<br>21	Rigid	0.719<br>0.254	**0.117**	0,063	0.176
TV	10<br>42	Rigid	0.719<br>0.318	**0.087**	0,125	0.176
TV	21<br>42	Rigid	0.254<br>0.318	**0.613**	0,625	0.613
TV	10<br>21	Semi-rigid	0.347<br>0.002	0.118	**0.156**	0.234
TV	10<br>42	Semi-rigid	0.347<br>0.102	**0.280**	0.438	0.280
TV	21<br>42	Semi-rigid	0.002<br>0.102	0.087	**0.156**	0.234
TV	10<br>21	Flexible	0.824<br>0.993	**0.000**	0,063	0.000
TV	10<br>42	Flexible	0.824<br>0.521	**0.000**	0,063	0.000
TV	21<br>42	Flexible	0.993<br>0.521	**0.340**	0,438	0.340

h) Time-series comparisons, BV/TV

Outcome measure	Day	Stiffness	Shapiro-Wilks (normality)	equal variance ttest	Wilcoxon	final adj *p*-value Hochberg
BV/TV	10<br>21	Rigid	0,150<br>0,871	**0.004**	0,063	0.006
BV/TV	10<br>42	Rigid	0,150<br>0,121	**0.001**	0,063	0.003
BV/TV	21<br>42	Rigid	0,871<br>0,121	**0.021**	0,063	0.021
BV/TV	10<br>21	Semi-rigid	0,178<br>0,487	**0.008**	0,031	0.024
BV/TV	10<br>42	Semi-rigid	0,178<br>0,046	0.001	**0.031**	0.047
BV/TV	21<br>42	Semi-rigid	0,487<br>0,046	0.051	**0.063**	0.063
BV/TV	10<br>21	Flexible	0,978<br>0,144	**0.035**	0,063	0.035
BV/TV	10<br>42	Flexible	0,978<br>0,181	**0.000**	0,063	0.000
BV/TV	21<br>42	Flexible	0,144<br>0,181	**0.005**	0,063	0.008

i) Time-series comparisons, TMD

Outcome measure	Day	Stiffness	Shapiro-Wilks (normality)	equal variance ttest	Wilcoxon	final adj *p*-value Hochberg
TMD	10<br>21	Rigid	0.006<br>0.777	0.841	**0.625**	0.625
TMD	10<br>42	Rigid	0.006<br>0.866	0.052	**0.125**	0.188
TMD	21<br>42	Rigid	0.777<br>0.866	**0.001**	0.063	0.003
TMD	10<br>21	Semi-rigid	0.119<br>0.26	**0.029**	0.063	0.029
TMD	10<br>42	Semi-rigid	0.119<br>0.257	**0.000**	0.031	0.000
TMD	21<br>42	Semi-rigid	0.26<br>0.257	**0.000**	0.031	0.000
TMD	10<br>21	Flexible	0.401<br>0.239	**0.329**	0.625	0.329
TMD	10<br>42	Flexible	0.401<br>0.972	**0.144**	0.188	0.216
TMD	21<br>42	Flexible	0.239<br>0.972	**0.000**	0.063	0.000

j) Time-series comparisons, TMC

Outcome measure	Day	Stiffness	Shapiro-Wilks (normality)	equal variance ttest	Wilcoxon	final adj *p*-value Hochberg
TMC	10<br>21	Rigid	0.363<br>0.491	**0.000**	0.063	0.000
TMC	10<br>42	Rigid	0.363<br>0.875	**0.000**	0.063	0.000
TMC	21<br>42	Rigid	0.491<br>0.875	**0.011**	0.063	0.011
TMC	10<br>21	Semi-rigid	0.920<br>0.002	0.002	**0.031**	0.031
TMC	10<br>42	Semi-rigid	0.920<br>0.716	**0.001**	0.031	0.003
TMC	21<br>42	Semi-rigid	0.002<br>0.716	0.024	**0.031**	0.031
TMC	10<br>21	Flexible	0.463<br>0.480	**0.004**	0.063	0.004
TMC	10<br>42	Flexible	0.463<br>0.810	**0.000**	0.063	0.000
TMC	21<br>42	Flexible	0.480<br>0.810	**0.002**	0.063	0.003

Figure S1:

A. Positive control

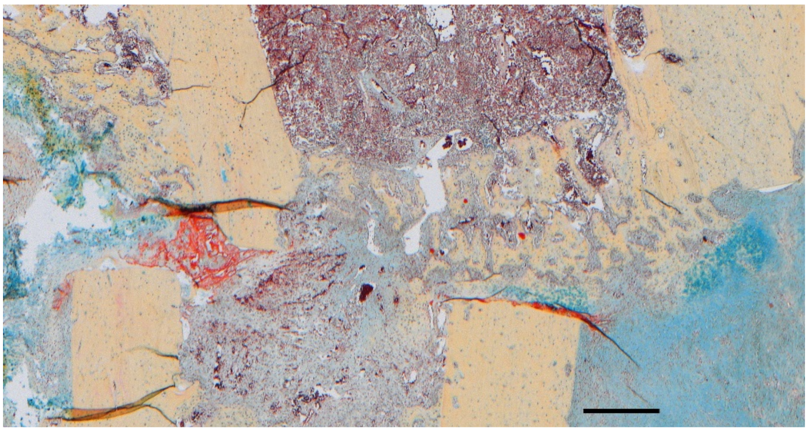

B. Negative control

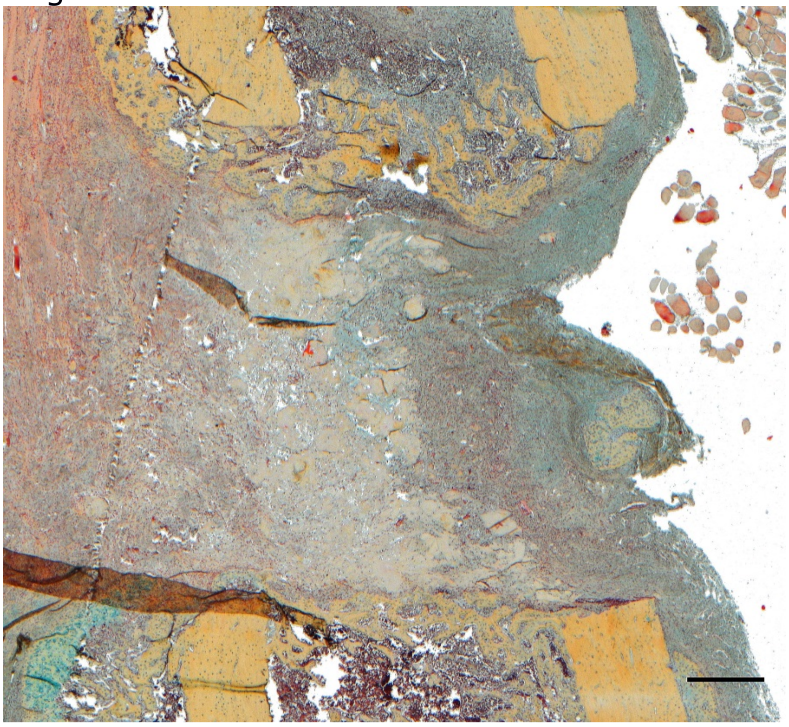

Figure S1. Representative histology of the control groups at day 14 post-operation. (A) Positive control (1mm osteotomy, top). The periosteal callus was characterized by woven bone with cartilage islands and some hematoma residues around the osteotomy ends. The endosteal callus separated the medullary canal tissue from the osteotomy gap that was filled with a proliferative tissue matrix. **(B) Negative control (untreated 5mm defect, bottom).** The defect site was filled with fibrous connective tissue and hematoma residues. Furthermore, residues of the absorbable collagen sponge were still detectable (Histology: Movat Pentachrome staining, scale bars: 500 µm).

Figure S2:

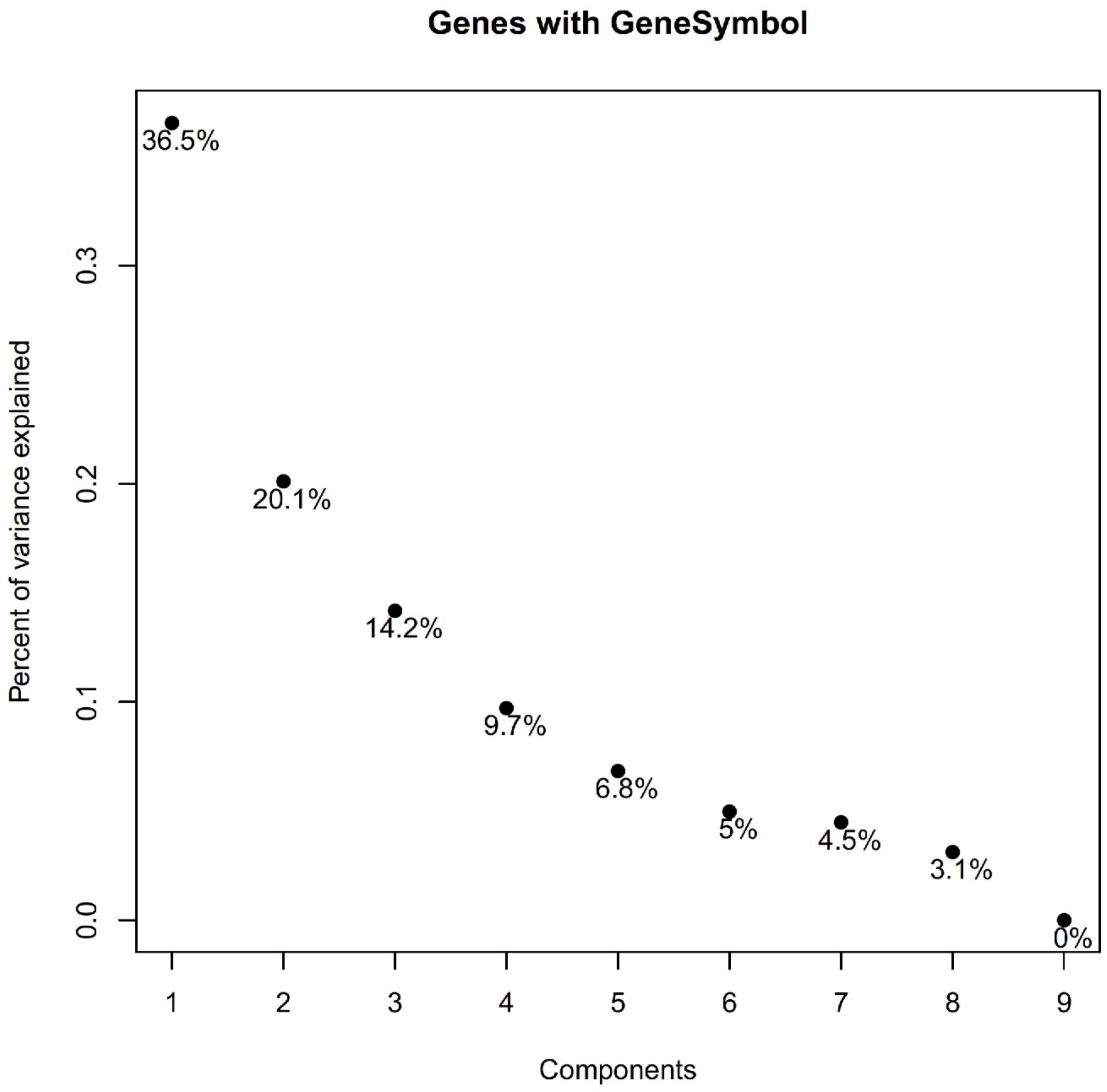

Figure S2. Singular value decomposition of gene expression data. Gene expression data of all entities annotated to a gene symbol were analyzed by singular value decomposition. The top four components explain more than 80 % of variance of gene expression (36.5+20.1+14.2+9.7=80.5).

Figure S3:

ID	name	Study term	Pop term	p.adjusted
GO:0031012	extracellular matrix	13	397	3.73E-10
GO:0043292	contractile fiber	9	206	4.94E-05
GO:0003012	muscle system process	9	339	0.007

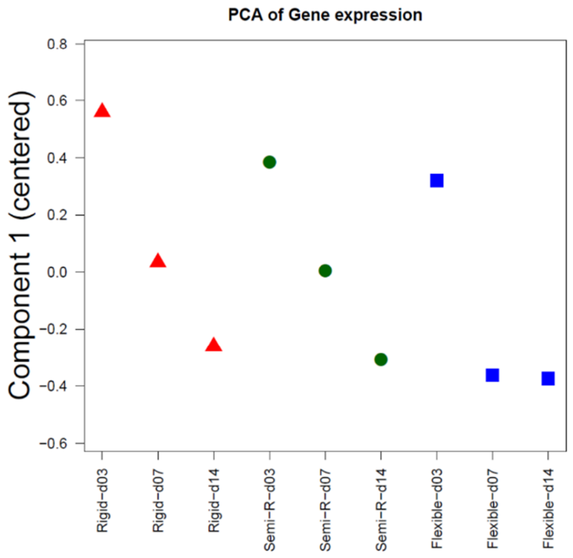

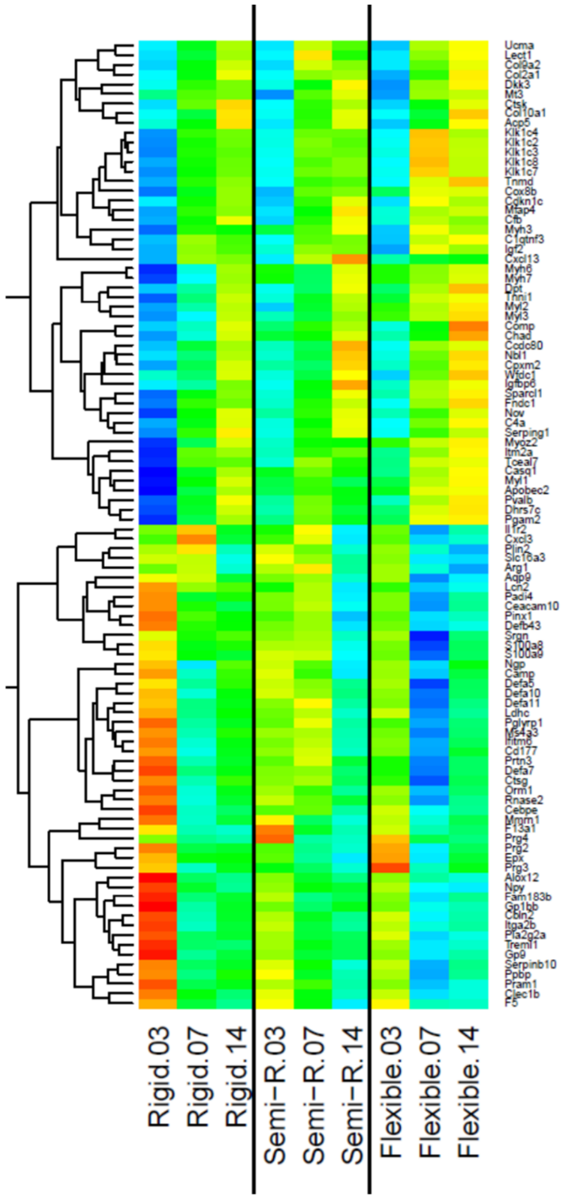

ID	name	Study term	Pop term	p.adjusted
GO:0002376	immune system process	22	2196	1.60E-07
GO:0009607	response to biotic stimulus	16	884	2.56E-04
GO:0050817	coagulation	8	178	8.14E-04
GO:0051704	multi-organism process	16	1883	0.001
GO:0005615	extracellular space	23	1419	0.003
GO:0009605	response to external stimulus	21	1999	0.013
GO:0070488	neutrophil aggregation	2	3	0.036

Figure S3. SVD – Component 1. Component 1 is the strongest component influencing variance of gene expression data. It explains 36.5 % of variance (cf. Suppl. Fig. S2). Component 1 captures gene regulation over time and comprises time dependent upregulation as well as time-dependent downregulation (red triangles: Rigid group, green dots: Semi-rigid group, blue squares: Flexible group). For each direction of regulation, top 50 genes annotated to a GO term were selected. In total top 100 genes were scaled to a mean of 0 and variance of 1 and subjected to hierarchical clustering (right). GO analyses were performed with the Parent-Child-Intersection (PCI-method) of Ontologizer. Terms with Bonferroni-adjusted $p < 0.05$ are displayed for genes of the corresponding cluster (top: upregulated genes, bottom: downregulated genes). Although the overall pattern is very similar, some differences between experimental groups can be observed. Upregulated genes are associated with '*extracellular matrix*' and '*contractile fiber*'. Products of downregulated genes are localized in the '*extracellular space*' and involved in '*immune system process*'-es.

Figure S4:

ID	name	Study term	Pop term	p.adjusted
GO:0002376	immune system process	31	2196	1.94E-18
GO:0001775	cell activation	15	876	1.27E-05
GO:0070661	leukocyte proliferation	9	303	0.006
GO:0098552	side of membrane	9	499	0.020
GO:0048872	homeostasis of number of cells	10	281	0.028
GO:0017171	serine hydrolase activity	7	217	0.038

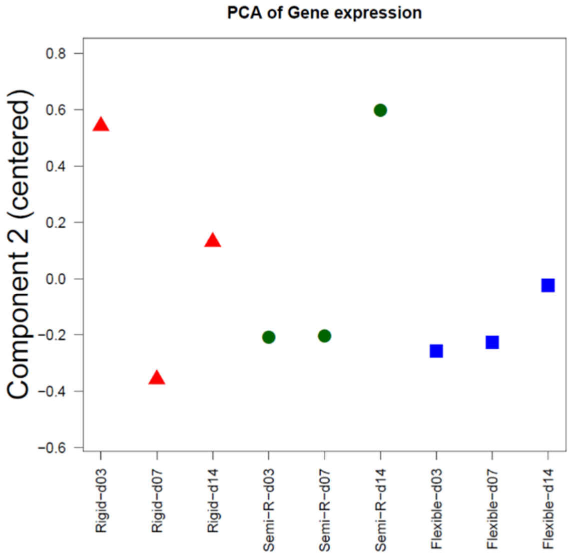

ID	name	Study term	Pop term	p.adjusted
GO:0043292	contractile fiber	16	206	1.63E-10
GO:0003012	muscle system process	11	339	1.74E-05
GO:0061061	muscle structure development	11	610	1.82E-04
GO:0008092	cytoskeletal protein binding	13	811	0.012
GO:0050879	multicellular organismal movement	4	45	0.025

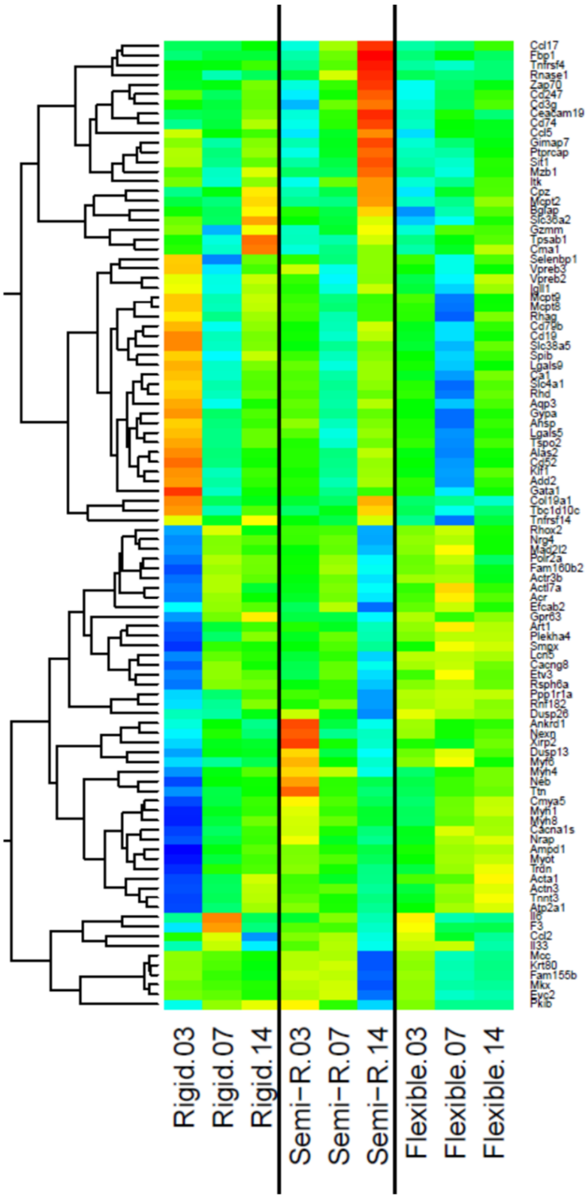

Figure S4. SVD – Component 2. Component 2 explains about 20 % of variance (cf. Suppl. Fig. S2). Expression levels are high (top of heatmap) and low (bottom of heatmap) in the rigid group at day 3 and show strong regulation in the semi-rigid group at day 14. Genes that contribute to component 2 are annotated to immunological processes and terms related to cellular contraction.

Figure S5:

ID	name	Study term	Pop term	p.adjusted
GO:0043292	contractile fiber	13	206	7.59E-08
GO:0003012	muscle system process	10	339	2.03E-06
GO:0008092	cytoskeletal protein binding	14	811	2.04E-04
GO:0061061	muscle structure development	12	610	0.002
GO:0030029	actin filament-based process	10	611	0.006
GO:0031432	titin binding	4	9	0.011

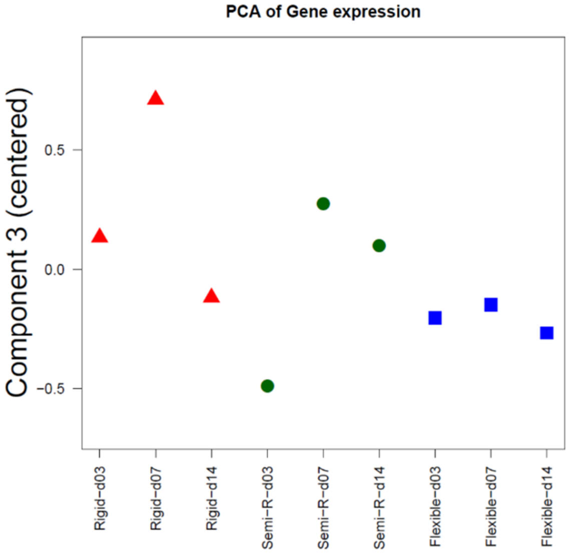

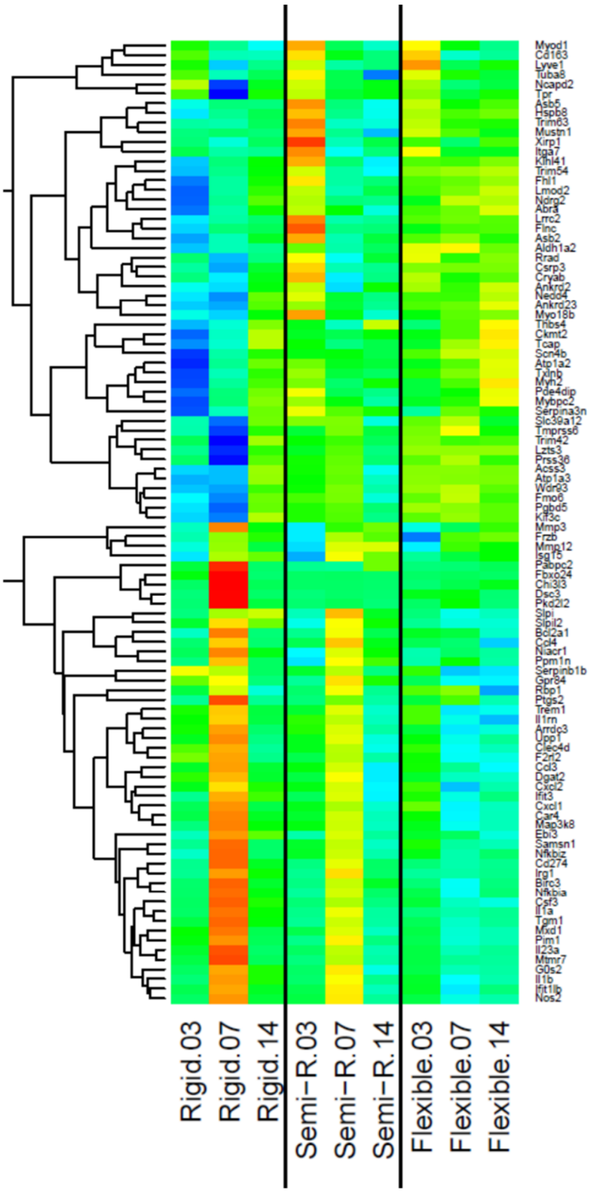

ID	name	Study term	Pop term	p.adjusted
GO:0002376	immune system process	27	2196	3.35E-12
GO:0040011	locomotion	15	1495	5.61E-04
GO:0005125	cytokine activity	10	216	0.001
GO:0051704	multi-organism process	16	1883	0.002
GO:0034097	response to cytokine	15	541	0.003
GO:0001906	cell killing	5	111	0.008
GO:0009607	response to biotic stimulus	15	884	0.009
GO:0001816	cytokine production	12	604	0.012
GO:0005126	cytokine receptor binding	10	284	0.015
GO:0006952	defense response	22	1336	0.023
GO:0072593	reactive oxygen species metabolic	7	235	0.030

Figure S5. SVD – Component 3. Component 3 explains about 14 % of variance (cf. Suppl. Fig. S2). From the point of view of the experimental setup, genes contributing to this component are highly interesting as they reflect the three different fixator stiffness groups. Strongest induction and downregulation was observed at day 7 in the rigid group, medium regulation in the semi-rigid group and rather unaltered expression levels were observed in the flexible group. This signature is more obvious in the top 50 genes with upregulation at day 7 (bottom of heatmap). These genes are annotated to terms related to the immune system and *'cytokine activity'* as well as *'cytokine production'*. Genes with opposite regulation pattern are related to the *'contractile fiber'* apparatus and *'actin-filament based process'*.

Figure S6:

ID	name	Study term	Pop term	p.adjusted
GO:0031012	extracellular matrix	12	397	4.11E-08
GO:0001503	ossification	14	379	5.63E-06
GO:0031214	biomineral tissue development	8	128	0.020
GO:0016787	hydrolase activity	18	2413	0.038
GO:0043062	extracellular structure organizatior	6	213	0.049

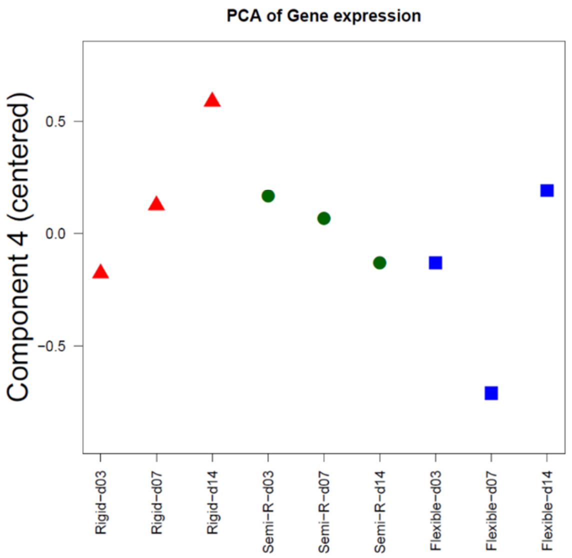

n.s.

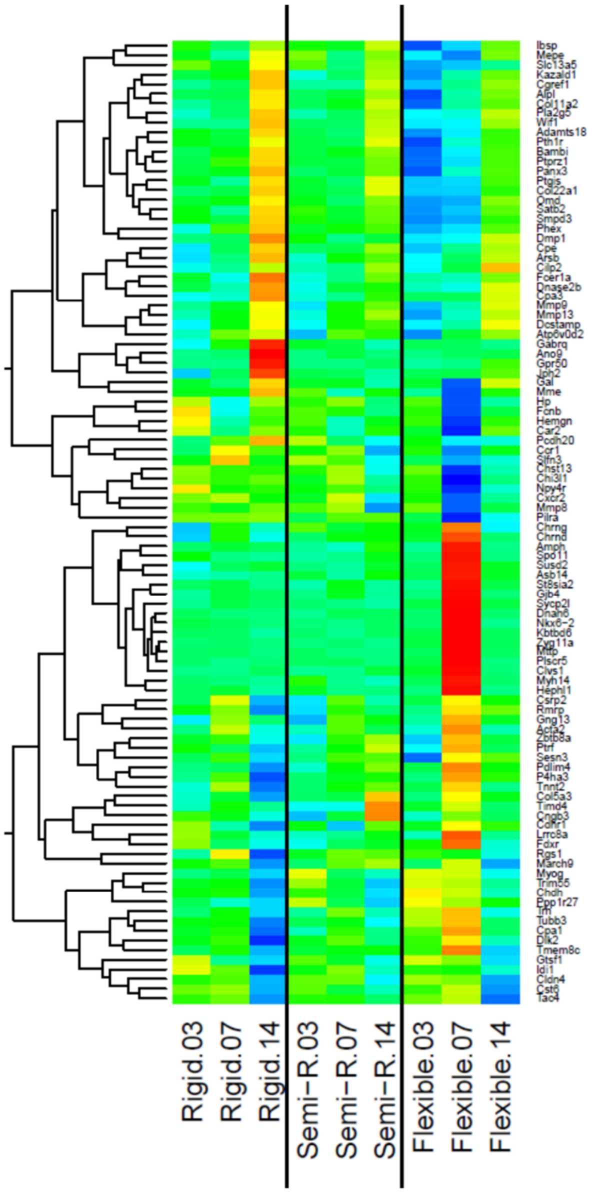

Figure S6. SVD – Component 4. Component 4 explains about 10 % of variance (cf. Suppl. Fig. S2). Although the overall expression pattern of genes contributing to this component is very different between experimental groups, hierarchical clustering reveals that there are to major blocks with differential regulation in the flexible group at day 7 and additional clusters that show differential expression related to gene regulation in the other two groups. Of note, 14 of the upper top 50 genes are annotated to '*ossification*' where induction is obviously delayed in the flexible group. GO analysis of genes localized in the lower part of the heatmap revealed no significant GO term (n.s.).

Figure S7:

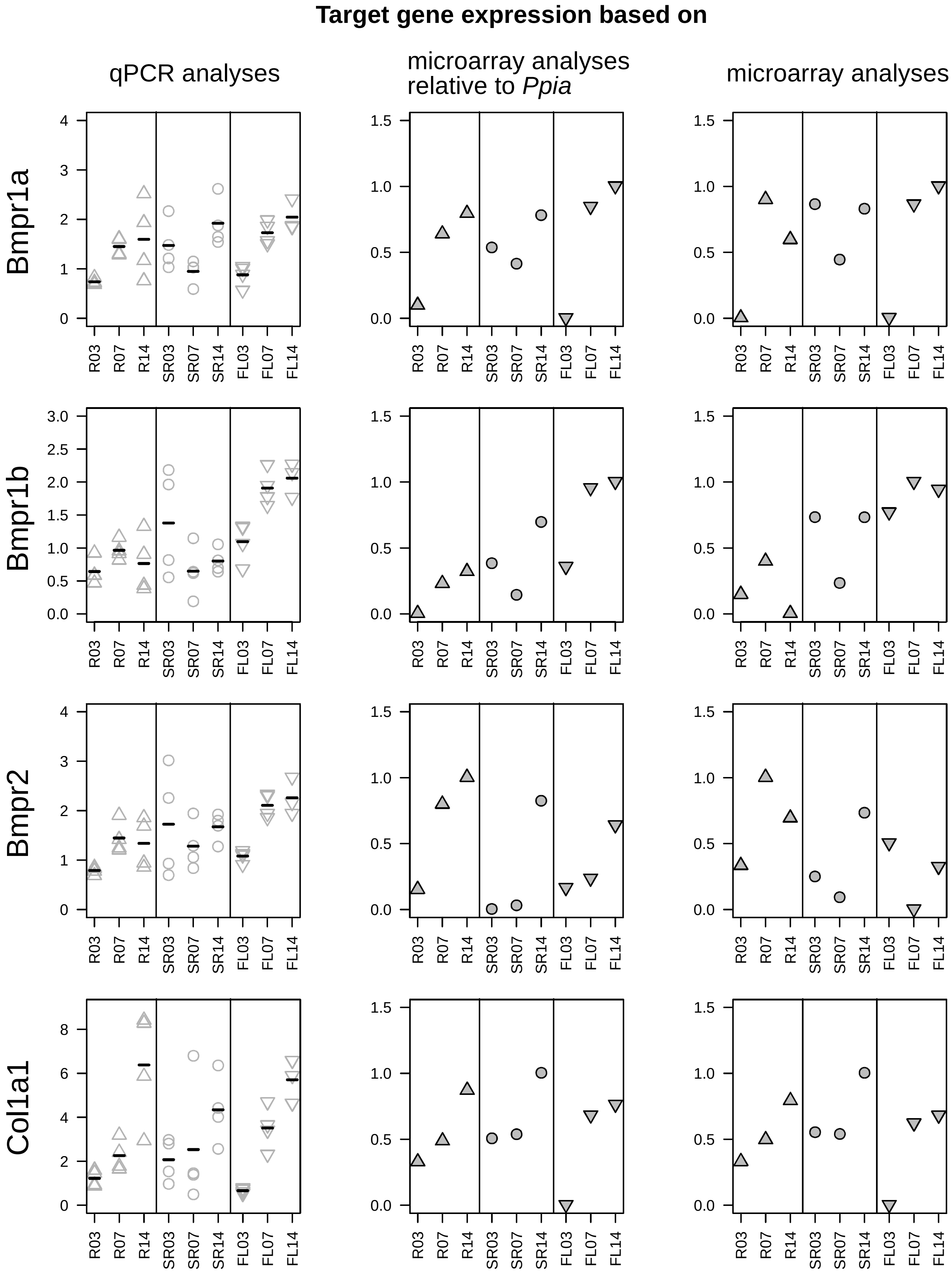

--- *continued next page* ---

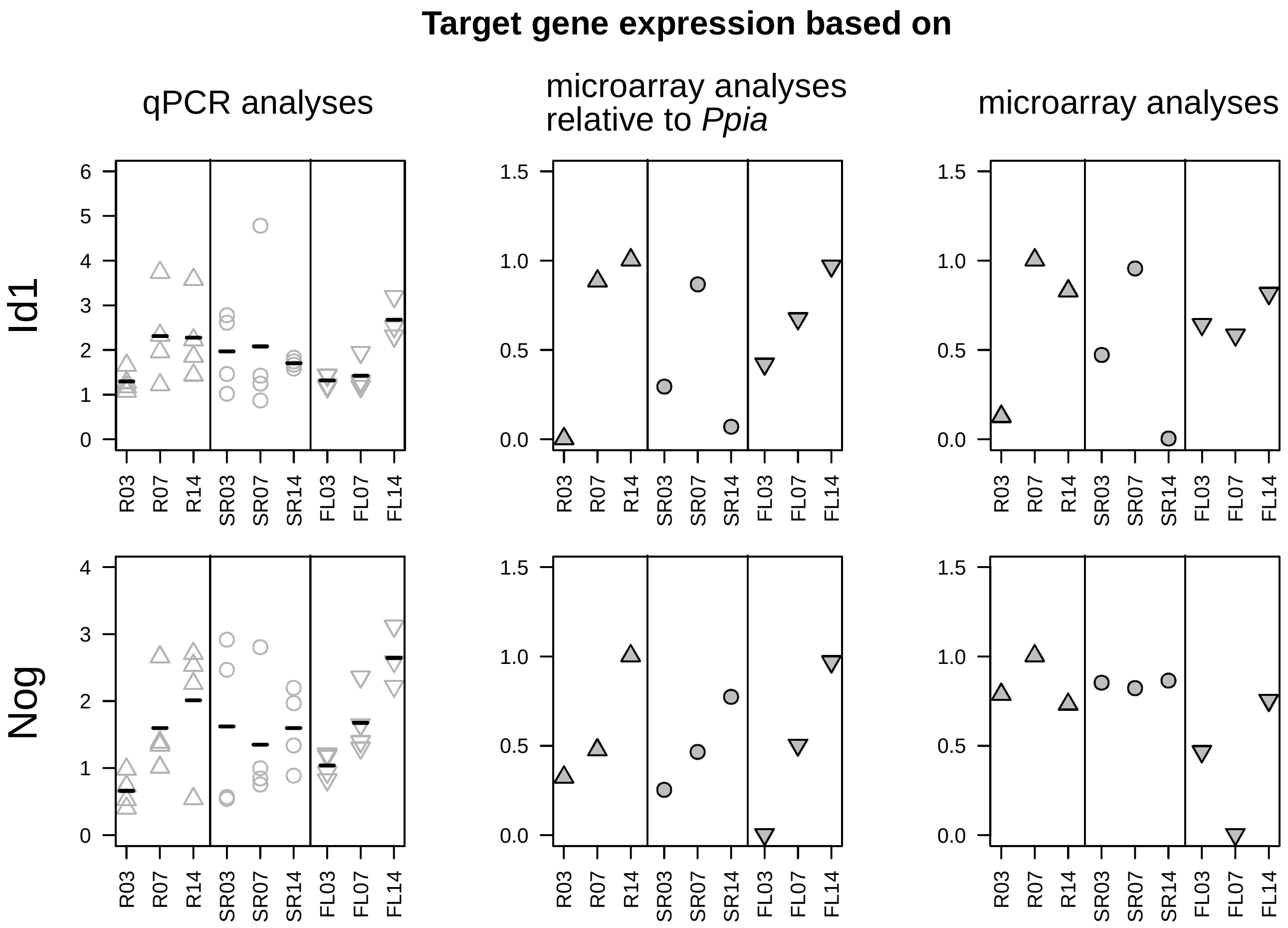

Figure S7. Expression of candidate genes analyzed by qPCR on microarrays. Left column displays expression values as determined by qPCR and shown in Figure 3 (R03 corresponds to Rigid group at day 3, SR. Semi-rigid group, FL. Flexible group, 07. day 7, 14. day 14). Middle column shows target gene expression relative to *Cyclophylin A* (*Ppia*) which was used as endogenous reference gene in qPCR analyses. These values are based on microarray data. Right column indicates expression values of these genes as determined by microarray analyses. (Middle and Right) Expression values derived from microarray analyses were rescaled to a minimum of zero and a maximum of one. Expression of *Bmpr1a*, *Bmpr1b*, *Col1a1* and *Id1* is very similar over all quantification approaches while expression of *Nog* displays changes in expression pattern dependent on the quantification method. Observed differences could be explained by differential expression of the reference genes used for qPCR analyses or limitations of determination of low level expression (running into background) when using microarrays.

Figure S8:

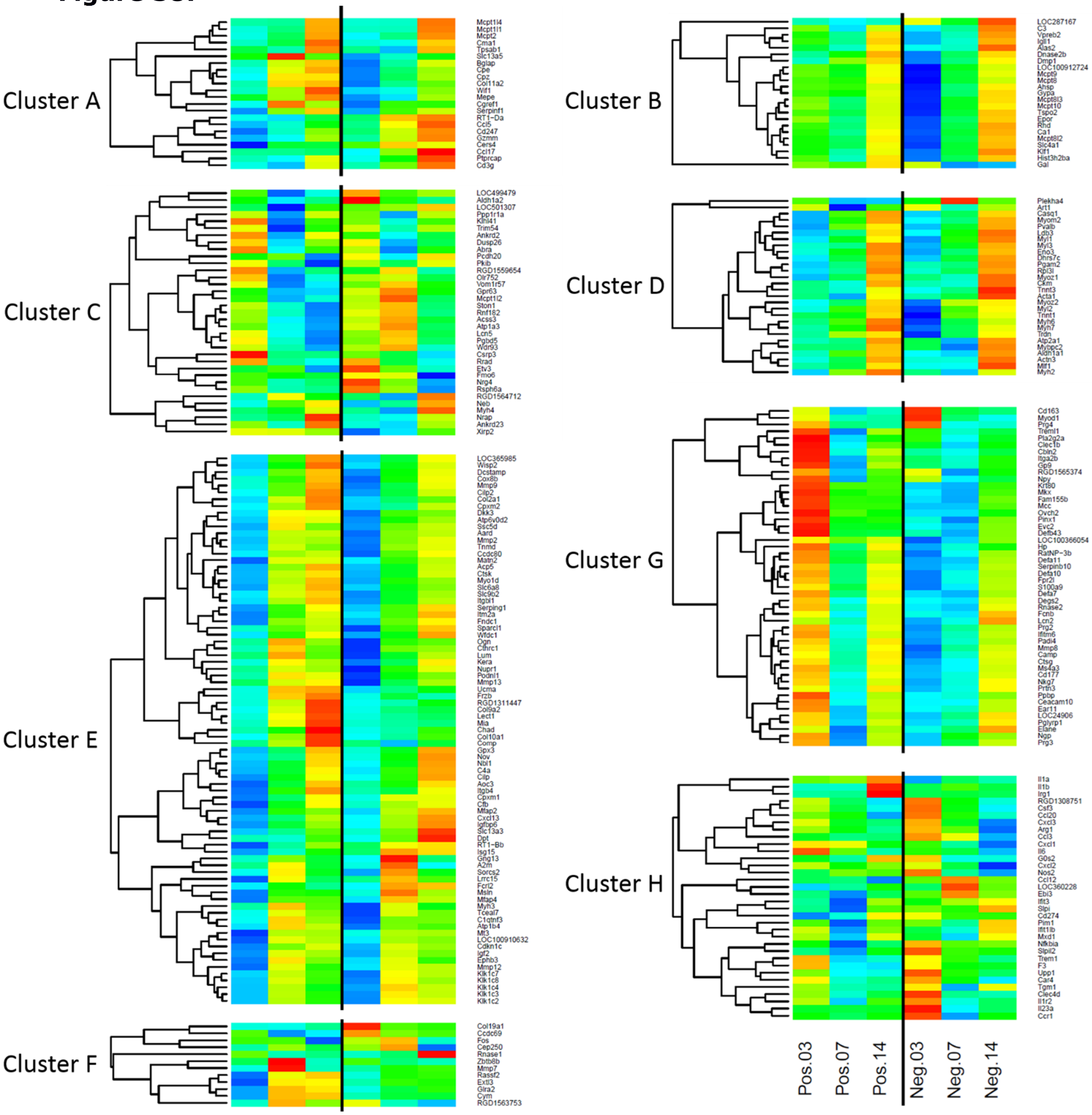

Figure S8. Expression pattern of the 285 high-variance genes in control groups. Genes of each cluster derived from Figure 5 were subjected to hierarchical clustering. The majority of these genes are differentially expressed in the control groups. Regarding genes from cluster A, *Bglap*, *Col11a2*, and *Mepe* display increasing expression levels in the positive, but not in the negative control where several cytokines are more highly expressed at later time points. Genes derived from cluster B including *Dmp1* have lowest expression levels in the negative control at day 3. Expression levels of genes from cluster C

mainly decrease in the positive, but increase in the negative control. A larger group of the genes from cluster D (*Myh6*, *Myh7*, *Myl2*, *Myoz2*, *Tnnt1*, and *Trdn*) display lower expression levels in the negative control. Regarding cluster E, similar to the pattern in the experimental groups increasing expression over time can be observed in the control groups. In the positive control at day 14 there are highest expression levels of the chondrocyte marker genes *Comp*, *Col10a1*, *Chad*, *Mia*, *Lect1* (chondromodulin), and *Col9a2*. As in the experimental groups, the majority of genes from cluster G shows an intermittent drop of expression at day 7 in the positive control while there is a slight induction in the negative control at day 14. The anti-inflammatory receptor *Cd163* and the myogenic factor *Myod1* show high expression levels in the negative control at day 3. Several cytokines from cluster H display higher expression levels in the negative control, especially at day 3.

Figure S9:

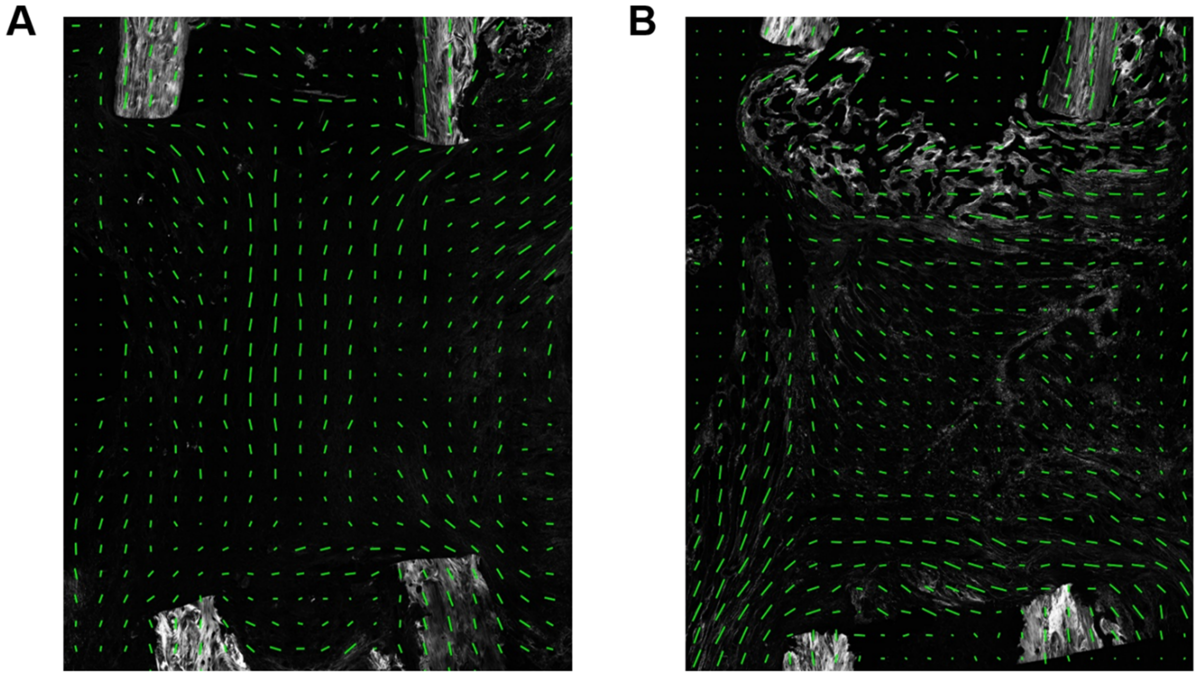

Figure S9. Second-harmonic generation (SHG) microscopy. (A) Flexible group at day 14. Fiber orientation in the middle of the defect runs in parallel to the bone axis (picture of a different animal of the flexible group, cf. Figure 6A). Overall, fiber orientation within the defect site resembles the shape of a sandglass. **(B) Semi-rigid group at day 14.** Fiber orientation at the margins of the fracture site runs in parallel to the bone axis while perpendicular fibers seal the medullary canal (picture of a different animal of the semi-rigid group, cf. Figure 6B). Organization of the fracture site is reminiscent of a crate or bowl shaped shelter. **(A, B)** Structures were visualized by SHG because collagen fiber orientation could not be visualized by Sirius Red staining and polarization microscopy. For SHG analyses, we selected sections without mineralized tissue. In the rigid group, all sections obtained by histology at day 14 contained significant amounts of mineralized tissue. Therefore, SHG was not applicable.

Figure S10:

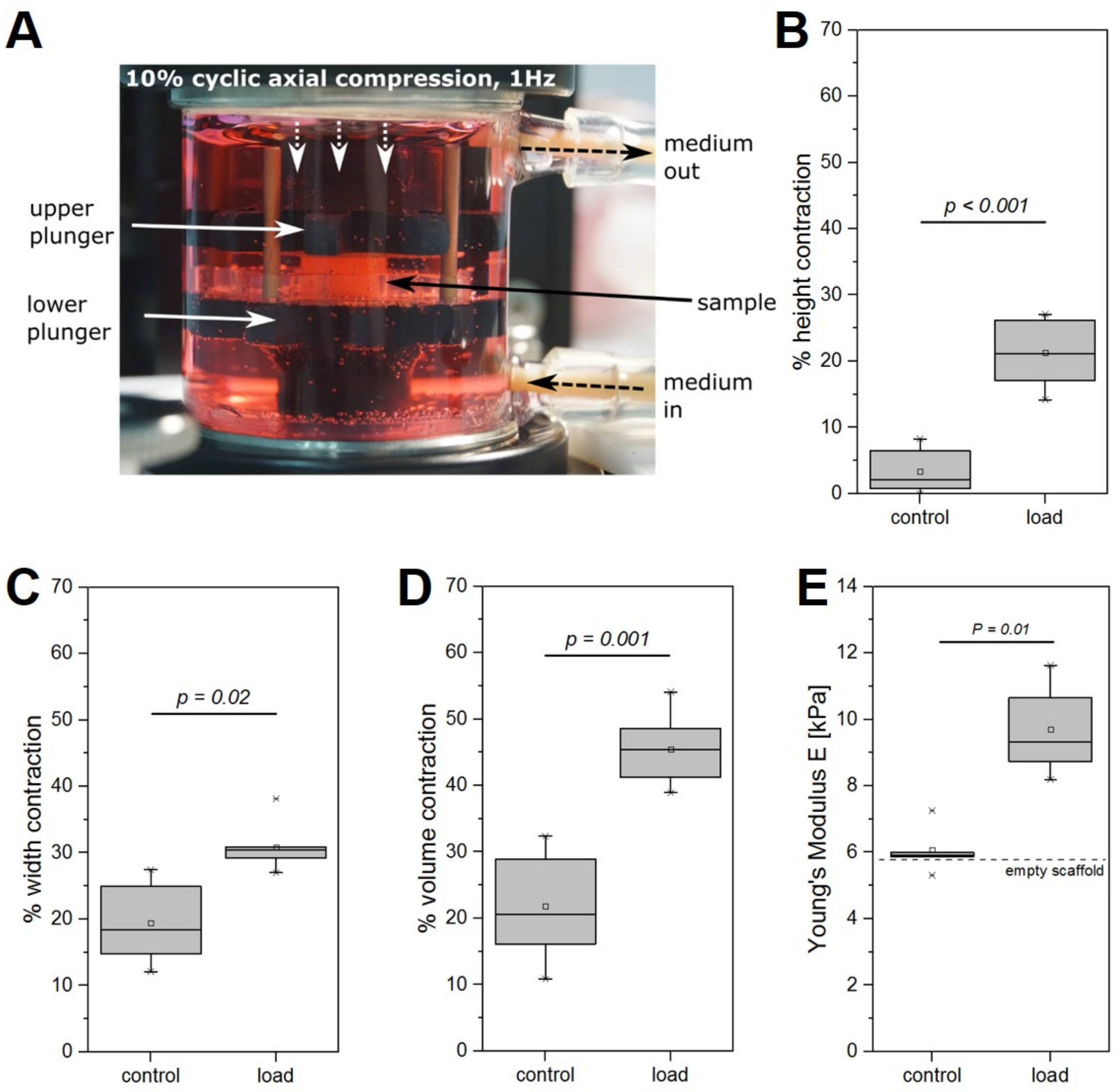

Figure S10. Bioreactor-based analysis of load-induced ECM contraction. (A) Custom-made mechano-bioreactor system used to apply 3 h cyclic monoaxial compression (f=1.0 Hz, 2A=300 µm ≙ 10 % scaffold height) and 5 h resting consistently repeated over one week. During resting phase this system automatically readjusts sample position to ensure proper mechanical stimulation. **(B) Significant height contraction upon loading.** Height refers to change of the scaffold height in parallel to loading/compression. **(C) Significant width contraction upon loading.** Width refers to change of the scaffold width, which is perpendicular to loading/compression. **(B, C)** Both parameters were combined for calculation of volume contraction as described in supplementary methods and shown in **(D) Significant difference in volume contraction upon loading. (B-D)** *n*=8 per group. Of note, the scaffolds used in these analyses have elastic mechanical properties. Therefore, height contraction is not a pure consequence of squeezing the scaffolds, which in turn would result in an increase but not decrease of the scaffold width. **(E)** Sample contraction leads to a significant increase in stiffness/rigidity as determined by Young's modulus (*n*=5 per group).

Supplementary methods

Cells, culture medium and seeding

Human fibroblasts were used in passages 6–9, expanded in Dulbecco's modified Eagle's medium (DMEM, #14965-039; Gibco, Life Technologies) supplemented with 10 vol% fetal bovine serum (FBS, #S0615; Biochrom AG), 1 vol% penicillin/streptomycin (#A2213; Biochrom AG), and 1 vol% nonessential amino acids (#K0293; Biochrom AG) in a humid incubator with 5 % CO_2. For bioreactor experiments FBS was reduced to 2 vol%, and 1.36 mM ascorbic acid was added to foster collagen formation. After expansion, cells were trypsinized and a cell concentration of 7500 cells/µL was adjusted. Collagen scaffolds (3 mm height, 5 mm diameter, Matricel GmbH) were seeded by dipping into the suspension and placed into an empty 12 well plate for one hour to allow cell adhesion to the scaffold walls, washed by immersion in medium to remove non-adherent cells, and placed in a medium-filled well overnight.

Bioreactor cultivation and mechanical stimulation

Two cell-seeded scaffolds were transferred into the custom-made mechano-bioreactor system [38], incubated overnight, then subjected to a sequence of 3 hours cyclic monoaxial compression (f = 1.0 Hz, 2A = 300 µm_≙ 10 % scaffold height) and 5 hours resting consistently repeated over one week. During resting phases (at 2.5 h), sample position was automatically readjusted by applying sinusoidal oscillation of the upper arm (f = 0.2 Hz, 2A = 100 µm) while the lower plunger moved up at a constant speed of 10 µm/s until a force magnitude 10 mN was detected. Repositioning ensured proper mechanical stimulation even if the sample contracted over time.

Scaffold contraction measurement and mechanical testing

Scaffold dimensions were assessed before and after bioreactor cultivation. Samples were scanned in both top and side view (resolution 1200 dpi, Epson Perfection #v200). Scaffold outlines were manually contoured to quantify cross sectional area and height of cylindrical samples. Relative sample contraction was calculated as ratio of scaffold measures after and before bioreactor cultivation. Additionally, samples were mechanically tested by monoaxial compression using a BOSE ElectroForce Mechanical Test Instruments TestBench system combined with a Model 31 Low load cell (Honeywell Corp.). Scaffolds were compressed in 3 cycles at 0.05 mm/s displacement speed over a distance of 10 % of the scaffold height calculated from scanned images before measurement. At 0 and 10 % strain, the position was kept constant for 30 seconds. Young's Modulus was calculated by linear fitting of stress-strain curves (range 4-10 %).
